# Recent Advances in Design of Gold-Based Catalysts for H_2_ Clean-Up Reactions

**DOI:** 10.3389/fchem.2019.00517

**Published:** 2019-08-07

**Authors:** Tatyana Tabakova

**Affiliations:** Institute of Catalysis, Bulgarian Academy of Sciences, Sofia, Bulgaria

**Keywords:** gold catalysts, hydrogen production, hydrogen purification, water–gas shift reaction, preferential CO oxidation

## Abstract

Over the past three decades, supported gold nanoparticles have demonstrated outstanding properties and continue to attract the interest of the scientific community. Several books and comprehensive reviews as well as numerous papers cover a variety of fundamental and applied aspects specific to gold-based catalyst synthesis, characterization by different techniques, relationship among catalyst support features, electronic and structural properties of gold particles, and catalytic activity, reaction mechanism, and theoretical modeling. Among the Au-catalyzed reactions targeting environmental protection and sustainable energy applications, particular attention is paid to pure hydrogen production. The increasing demands for high-purity hydrogen for fuel cell systems caused a renewed interest in the water–gas shift reaction. This well-known industrial process provides an attractive way for hydrogen generation and additional increase of its concentration in the gas mixtures obtained by processes utilizing coal, petroleum, or biomass resources. An effective step for further elimination of CO traces from the reformate stream after water–gas shift unit is the preferential CO oxidation. Developing highly active, stable, and selective catalysts for these two reactions is of primary importance for efficient upgrading of hydrogen purity in fuel cell applications. This review aims to extend the existing knowledge and understanding of the properties of gold-based catalysts for H_2_ clean-up reactions. In particular, new approaches and strategies for design of high-performing and cost-effective formulations are addressed. Emphasis is placed on efforts to explore appropriate and economically viable supports with complex composition prepared by various synthesis procedures. Relevance of ceria application as a support for new-generation WGS catalysts is pointed out. The role of the nature of support in catalyst behavior and specifically the existence of an active gold–support interface is highlighted. Long-term stability and tolerance toward start-up/shutdown cycling are discussed. Very recent advances in catalyst design are described focusing on structured catalysts and microchannel reactors. The latest mechanistic aspects of the water–gas shift reaction and preferential CO oxidation over gold-based catalysts from density functional theory calculations are noted because of their essential role in discovering novel highly efficient catalysts.

## Introduction

For over three decades, catalysis by gold continues to be a topic of special interest to stimulate research activities in discovering new properties and attractive applications of of gold-based catalysts. The scientific community is already well aware about the catalytic power of supported gold nanoparticles. Several books (Bond et al., 2006; Avgouropoulos and Tabakova, 2013; Ma and Dai, 2014; Mishra, 2016; Pratti and Villa, 2016) and special issues in reputable journals (*Applied Catalysis A: General*, vol. 291, 2005; *Chemical Society Reviews*, vol. 37, 2008; two SI in Catalysts: “Gold Catalysts”, 2011 and “New Trends in Gold Catalysts”, 2013), many chapters and reviews (Bond and Thompson, 1999; Haruta, 2002, 2003, 2004; Hashmi, 2007; Hutchings, 2007; Yan et al., 2007; Carabineiro and Thompson, 2010; Gong, 2012; Hutchings and Edwards, 2012; Barakat et al., 2013; Guan et al., 2015; Ma et al., 2015), and a huge number of papers have tackled fundamental and applied aspects related to gold-based catalyst preparation methods and their impact on gold particle size, shape, and oxidation state, role of the nature of support and support effects, relationship between electronic and structural properties of the gold-based catalysts, and catalytic performance in various reactions. Many research efforts have been focused on elucidating a surprisingly high effectiveness of supported gold nanoparticles in a wide variety of processes. A breakthrough in the perception of the poor catalytic activity of gold was made at the end of the 1980s. Haruta et al. (1987) discovered an extremely high CO oxidation activity of gold nanoparticles supported on transition metal oxides at, or even below, room temperature. At the same time, Hutchings revealed the catalytic potential of gold nanoparticles for hydrochlorination of acetylene to vinyl chloride (Hutchings, 1985). These groundbreaking works encouraged many scientists to be involved in the attractive research area of gold catalysts that opened up new opportunities for the catalysis community. During the next years, an impressive growth of scientific investigations concerning catalysis by gold occurred and totally changed the opinion of the catalytic resistance of gold. Now, without any doubt, we can affirm that gold loses its nobility when occurring at the nanometer length scale. Corma and Garcia (2008) explained in a perfect way this phenomenon, describing “gold catalysis as a paradigmatic example of those properties that are only observed in nanoparticles and can disappear completely as the particle size grows into the micrometric scale.”

**Figure 1:**
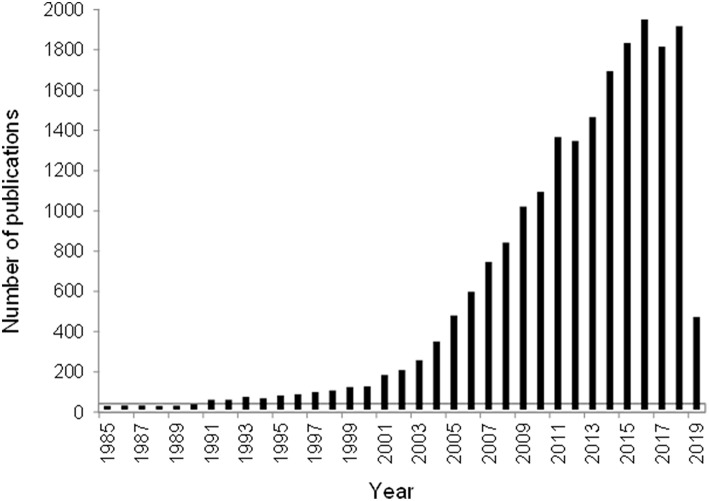
Number of publications on the topic “Gold catalysts” for the period 1985–March 2019 according to the database Web of Science.

An undisputed evidence of ever-growing interest expressed as the number of publications per year on the topic of “gold catalyst” (based on Scopus or WoS database) was illustrated in several reviews (Gutiérrez et al., 2011; Centeno et al., 2016; Genty et al., 2017). Although there were some fluctuations after 2011, an increasing trend of the published papers was also registered by the end of March 2019 (Figure 1). This observation gives a reason to admire the efforts of Guest editors for celebrating the success of catalysis by gold by editing this special issue. In particular, one of the aims, namely, contribution of gold-based catalysts to the development of greener and more sustainable societies, is appealing.

Among the vast literature on attractive catalytic performances of gold-based catalysts for reactions, targeting environmental protection and sustainable energy applications, special attention is given to pure hydrogen production. Very recently, a comprehensive review highlighted the major role that hydrogen could provide for efficient transition toward low-carbon economy by 2050 (Staffell et al., 2019). Hydrogen-powered vehicles would significantly contribute to decarbonizing the transport sector. In this context, developing highly active, stable, and selective catalysts is of primary importance for efficient upgrading H_2_-rich gas streams for fuel cell applications. This review aims to extend existing knowledge and understanding of the properties of gold-based catalysts for H_2_ clean-up reactions, focusing on the water–gas shift (WGS) reaction and preferential CO oxidation in hydrogen-rich gas stream (PROX). New approaches and strategies for design of well-performing and cost-effective formulations are discussed. Particular emphasis is given on the efforts to study appropriate and economically viable supports with complex composition prepared by different synthesis procedures. Gold on ceria-containing supports continues to attract research interests, and recent progress for the WGS and PROX reactions is summarized. Long-term stability and tolerance toward start-up/shutdown operations are addressed. Very recent advances in catalyst design are described focusing on powder catalysts with a brief comment on the developments of structured catalysts and microchannel reactors. Latest mechanistic aspects of the WGS and PROX reactions over gold-based catalysts from density functional theory (DFT) calculations are noted because of their substantial role in discovering novel catalysts with efficient catalytic performance.

## General Overview of the Literature on Gold-Catalyzed WGS Reaction

Among the gold-catalyzed reactions, pure hydrogen production has received considerable attention aimed at improving the quality of life by environmental pollutant abatement and sustainable energy production. Hydrogen energy is a very attractive research area with growing relevance to future energy challenges. The ever-increasing CO_2_ emission due to fossil fuel consumption accounts for over a half of the enhancement of the greenhouse effect that causes global warming. This has resulted in an increased demand of effective clean-up technologies and search for alternative fuels, their utilization being accompanied by toxic free emissions. Achieving a zero-waste emission by using clean energy sources and reducing greenhouse gas emissions would positively affect social and economic development and would contribute greatly to improve current environmental conditions. Hydrogen has several advantages as an energy carrier. Among all fuels, it has the highest energy content per unit mass, which is almost three times higher than that of gasoline. Moreover, hydrogen can offer economically viable, financially attractive, and socially beneficial solutions of the growing concerns about global warming and increasing world energy demand (Hydrogen Council, 2017).

Very recently, Baykara (2018) published an overview of available methods for hydrogen production focusing on the use of different sources like fossil fuels, in particular natural gas and coal, biomass, water, metal hydrides, H_2_S, and biological materials. Among them, emphasis was put on the use of fossil fuels, because it is based on well-established technologies that provide higher efficiency and a lower product cost, although there are existing concerns with depletion of fossil resources and CO_2_-induced climate change. A multistep process of hydrogen production includes syngas generation by reforming of hydrocarbons, produced from fossil fuel or renewable resources, followed by WGS reaction (CO + H_2_O ↔ CO_2_ + H_2_). Further decrease of CO content can be achieved by selective methanation, or preferential oxidation of CO, or both reactions. The WGS reaction has a long historical application, and today, it is already a well-established industrial process for generation of hydrogen and additional increase of its concentration in the gas mixtures obtained by processes utilizing coal, petroleum, or biomass sources. Several reviews have discussed developments and recent advances in WGS catalysis (Hinrichsen et al., 2008; Ratnasamy and Wagner, 2009; Deshpande and Madras, 2013). Reddy and Smirniotis (2015) outlined the importance of the WGS reaction and its application for hydrogen production in a comprehensive book. A historical background along with thermodynamic aspects of the reaction is presented. The book provides a detailed survey of different types of WGS reactors and catalysts including the effect of various parameters as composition, role of promoters, method of preparation, catalyst activation, etc. on catalyst performance. Pal et al. (2018) summarized the progress in the field of WGS catalysts, pointing out those that have been recently developed in order to achieve better catalytic efficacy and cost efficiency.

Presently, the emerging hydrogen economy demonstrates a very high potential of fuel cells to replace the internal combustion engine in vehicles and to supply power in stationary and portable devices due to their high-energy efficiency, cleanness, and fuel flexibility. Together, hydrogen and fuel cells represent a radically different approach to energy conversion. Roberts et al. consider that hydrogen and fuel cell technologies are very close to the commercial implementation at a meaningful scale and anticipate the realization within the end of this decade (Roberts et al., 2018). In this context, the WGS reaction attracted renewed interest owing to the increasing demands for high-purity hydrogen. The hydrogen-rich gas stream after WGS reaction contains typically 0.5–1 vol.% CO because of thermodynamic limitation of this reversible and moderately exothermic reaction. Due to the high sensitivity of platinum anode electrode in polymer electrolyte membrane (PEM) fuel cells toward even low CO levels, the concentration of CO should be reduced to below 10 ppm (below 100 ppm for CO-tolerant alloy anodes). An effective step for elimination of CO traces from the hydrogen-rich reformate gas stream after WGS unit is the PROX reaction. Ivanova et al. have described CO clean-up fundamentals in detail focusing on the current practice in industrial hydrogen production and highlighting the requirements that should be met by processes and catalysts for small-scale applications such as residential fuel cells or on-board hydrogen generators (Ivanova et al., 2013). Because of safety and technical constraints, it was shown that well-established commercial Cu/ZnO-based and Fe_2_O_3_-Cr_2_O_3_ based catalysts for large-scale hydrogen production are not suitable for fuel processors. These catalysts require special activation procedures before use; they are pyrophoric, intolerant to poisons, susceptible to oxidation and condensation. Additionally, some technical issues like small catalyst volume and low weight, reduced start-up time, stability under steady state and transient conditions impose new goals and challenges for design of WGS and PROX catalysts. Developing well-performing, stable, and selective catalysts for WGS and PROX reactions is of primary importance for efficient upgrading of hydrogen purity for fuel cell applications.

Rational design of advanced WGS catalysts is closely related to the understanding of the reaction mechanism. Based on extensive DFT calculations, Liu has demonstrated a possibility to gain fundamental understanding of the WGS reaction (Liu, 2013). In this review, the author provides guidance for developing improved catalysts based on mechanistic understanding, starting with simple metal surfaces and moving on to oxide-supported metallic nanoparticles, metal-supported oxide nanoparticles, and more complex mixed-metal oxides. It was concluded that oxide-supported metal (Au, Cu, Pt, Pd) catalysts manifested a very promising behavior for WGS reaction in small-scale applications.

Following the great success of Haruta's discovery of remarkably high CO oxidation activity of gold-based catalysts, Andreeva et al. (1996) presented the earliest report on applicability of oxide-supported gold catalysts for CO removal from hydrogen feed by the WGS reaction. The first summary of the results reported in the literature on the WGS reaction over gold-containing catalysts was published in 2002 (Andreeva, 2002). The effects of the preparation method and the nature of support on gold dispersion and WGS activity were discussed. The influence of gold presence on enhanced reducibility of the metal oxide support was commented. A few years later in an excellent review, Burch analyzed kinetic results for the WGS reaction on noble metal catalysts and demonstrated successful application of oxide-supported gold catalysts (Burch, 2006). A detailed comparison between WGS performance of Au- and Pt-based catalysts revealed that well-prepared gold catalysts exhibited a significantly higher activity than the platinum catalysts. This work addressed the crucial importance of the preparation method and variations in activation and testing conditions on the activity of the WGS catalysts. By critical assessment of the published data about structure of the active sites for the WGS reaction and numerous arguments on the possible reaction mechanism at low temperatures over supported noble metal catalysts, a “universal” model has been proposed. It is in good agreement with most of the available experimental and theoretical results and points out a key role of the reaction conditions on the nature of the active catalyst surface. In 2013, Tao and Ma summarized in a comprehensive review the progress in the development of gold catalysts for WGS reaction (Tao and Ma, 2013). This work evaluated different phases of research, starting from the first reports of high WCS activity of gold catalysts supported on reducible metal oxides and comparison with gold counterparts based on non-reducible carriers as well as with other WGS catalysts. Next stages were focused on efforts for understanding the impact of preparation methods on catalytic performance or development of novel gold catalysts with variations in chemical composition, morphology, or support structure. New experimental evidences were discussed to support deactivation mechanisms already examined by Burch. The authors critically analyzed the latest findings about the nature of active sites and reaction mechanisms by highlighting the important role of surface chemistry and structure of oxide-supported gold catalysts during WGS reaction by using various *in situ* techniques.

Ramirez Reina et al. (2014) presented the most exhaustive review in the field of WGS catalysis by gold under the attractive title: “Twenty years of golden future in the water–gas shift reaction.” The authors applied a very original way of collecting all the existing information from 1996 to 2014 by grouping in 5-year periods. They have thoroughly summarized and discussed within each period various important aspects of preparation, characterization, and application of gold-based WGS catalysts by emphasizing on the effect of support nature and gold–support interaction on catalyst behavior. Comparing gold and other noble metals, the authors underlined the benefits and disadvantages of the application of gold-based materials in the low-temperature WGS reaction.

The latest contribution to the gold-catalyzed low-temperature WGS reaction belongs to Carter and Hutchings who evaluate recent advances in the field (Carter and Hutchings, 2018). Alongside with a detailed description of the most active catalysts, considerable attention is placed on a very challenging issue: development of highly active and robust catalysts that do not deactivate on-stream under realistic reaction conditions. Recent computational and fundamental aspects are also discussed.

## Recent Achievements in the Field of Gold-Catalyzed WGS Reaction

### Preparation of Powder Catalyst: New Approaches for Improved Efficiency

**Figure 2:**
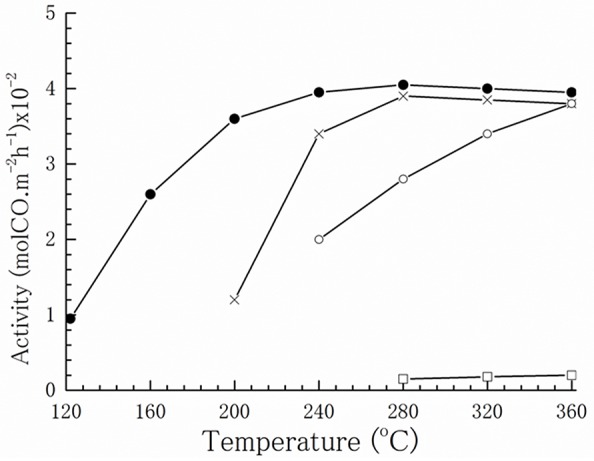
Temperature dependence of WGS activity: (●) Au/α-Fe_2_O_3_, (o) α-Fe_2_O_3_, (□) Au/Al_2_O_3_, and (x) CuO/ZnO/Al_2_O_3_. Reprinted with permission from Andreeva et al. (1996). Copyright Elsevier B.V.

Based on numerous studies, undoubtedly the exploration of appropriate supports is of key importance to prepare well-performing gold catalysts for the WGS reaction. The support may directly participate in the reaction or govern catalytic performance by affecting shape and gold particle size, gold–support interface interaction, and stabilization of structural/electronic properties of gold. Kinetic and operando FTIR measurements of the WGS reaction over gold nanoparticles of different average size supported on model Al_2_O_3_ and TiO_2_ provided evidence for a direct role of the support in water activation, while adsorption of CO and formation of CO_2_ and H_2_ took place on gold (Shekhar et al., 2012). Schubert et al. discussed the impact of support reducibility and oxygen storage capacity (Schubert et al., 2001). Gold on non-reducible supports, such as SiO_2_, Al_2_O_3_, or MgO, demonstrated lower activity in contrast to deposited gold on reducible materials that exhibited a significantly enhanced CO oxidation. A similar support effect was observed in the case of gold-catalyzed WGS reaction. Andreeva et al. (1996) were the first who compared WGS activity of gold nanoparticles supported on Fe_2_O_3_ and Al_2_O_3_. Despite the similarity of gold particle size on both supports (3.5 nm), a very low activity of Au/Al_2_O_3_ was observed, indicating a decisive role of support nature (Figure 2).

Sandoval et al. (2007) have studied Au/TiO_2_, Au/CeO_2_, Au/Al_2_O_3_, and Au/SiO_2_ and found a much higher WGS activity of gold nanoparticles on reducible supports (TiO_2_ and CeO_2_) than on Al_2_O_3_ and SiO_2_. Lenite et al. (2011) evaluated gold supported on CeO_2_ and (α-γ)/Al_2_O_3_ prepared through solution combustion synthesis and observed that alumina-supported gold catalysts attained only about 30% CO conversion. In this case, the authors pointed out an indirect role of the support allowing penetration of small gold particles (2–3 nm) into pores of 4 nm average size, thus negatively affecting access of CO and water. In a very recent review, Carter and Hutchings (2018) also stressed on support effects in WGS gold catalysts using reducible and non-reducible materials.

#### The Application of “Supported Approach”

**Figure 3:**
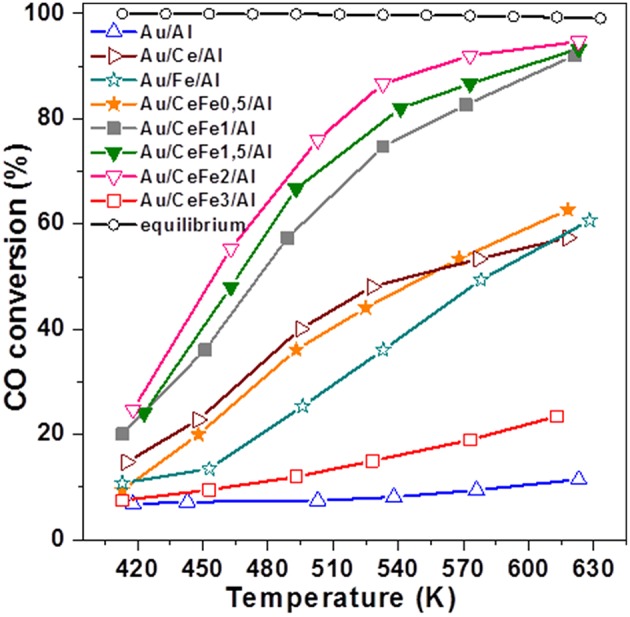
Temperature dependence of CO conversion over the studied catalysts. Reprinted from Reina et al. (2013a) with permission from the Royal Society of Chemistry.

During the last few years, Reina et al. (2013a) proposed a new strategy for development of efficient and economically viable gold-based catalysts for WGS reaction. Commercial γ-alumina has been modified by well-dispersed surface fraction of Ce–Fe mixed oxides (15 wt.%) and used as a support for gold-based catalysts. This synthesis approach allows maximizing the activity per mass of gold due to its high dispersion on the support surface, thus achieving both a greater interfacial area between the gold nanoparticles and alumina-supported mixed oxides and enhanced oxygen mobility. From an economic point of view, another advantage is a lower amount of ceria in the catalyst formulation as compared to Au/CeO_2_ (Odriozola et al., 2013). Ceria and iron oxide selection was properly considered based on the existing knowledge of their direct role in the WGS reaction. Since the earliest reports from the groups of Flytzani-Stephanopoulos (Fu et al., 2001) and Andreeva (Andreeva et al., 2002) about excellent WGS activity of Au/CeO_2_ at low temperatures, numerous studies have clarified how some specific features of ceria affect WGS performance of this kind of catalysts. These include nano-scaled particle size, surface area, surface structure, shape and crystal plane, and especially gold–ceria interface (Karpenko et al., 2007; Si and Flytzani-Stephanopoulos, 2008; Ta et al., 2012; Guan et al., 2015; Yi and Flytzani-Stephanopoulos, 2015; Centeno et al., 2016; Fu et al., 2019). Ceria became an attractive support for new-generation WGS catalysts owing to its ability to undergo a fast Ce^4+^ ↔ Ce^+^ transfer and to dissociate water on created oxygen vacancies. The key role of oxygen vacancies in facilitating the most difficult step in the WGS reaction, i.e., the dissociation of H_2_O, whatever redox or associative mechanism is operating, was experimentally explored (Rodriguez et al., 2007; Vindigni et al., 2011) and reviewed (Laguna et al., 2014). Iron oxide has also been reported as a suitable support for active gold-based WGS catalysts due to the redox transfer Fe^3+^ ↔ Fe^2+^ in Fe_3_O_4_ formed under WGS conditions (Andreeva et al., 1996). The benefits of iron-modified ceria in improving the CO oxidation activity of Au/FeO_x_/CeO_2_ (3.37 wt.% Fe) was ascribed to the presence of Fe^3+^/Fe^2+^ and Ce^4+^/Ce^3+^ redox couples affecting the existence of Au^+^/Au^0^ (Au^δ+^/Au^0^) redox couples (Penkova et al., 2009). Using impregnation or mechanochemical mixing, Ilieva et al. have prepared gold catalysts by deposition–precipitation on ceria modified by varying Fe_2_O_3_ content (5, 10, and 20 wt.% Fe_2_O_3_) (Ilieva et al., 2013). The ceria doping method significantly affected gold dispersion. The highest WGS activity of gold catalysts using a support prepared by mechanochemical mixing with lowest amount of hematite was related to available gold particles of smaller size and to enhanced hematite reducibility due to surface interaction between Fe and Ce cations. Being important, the Ce–Fe interaction was also discussed in the abovementioned work of Reina et al. (2013a). The WGS activity of Au/CeO_2_-Al_2_O_3_ increased as a function of the iron content (0.5–2.0 wt.%) and was significantly higher than that of the undoped sample. Activity improvement was attributed to a synergetic effect between ceria and iron oxide and their cooperative role in water activation, despite high gold dispersion and increased number of oxygen vacancies that also contributed to this (Figure 3). An overview of some characteristics of selected gold-based catalysts using supports prepared by “supported approach” and their WGS performance is presented in Table 1.

**Table 1:** Overview of some characteristics of selected gold-based catalysts using supports prepared by “supported approach” and their WGS performance.

**Catalyst**	**Composition wt. %**	**Gold content wt.%**	**Gold size nm**	**Synthesis method^**a**^**	**Synthesis method^**b**^**	**Reaction conditions**	**WGS activity^**c**^**	**References**
Au/FeO_x_-CeO_2_/Al_2_O_3_	Al_2_O_3_−81.2 CeO_2_−14.9 Fe_2_O_3_−1.72	2.17	4.0	DAE	IMP on Al_2_O_3_ (Sasol)	0.5 cm^3^; 3.4% CO, 25.0% H_2_O, 71.6% Ar; GHSV 4 000 h^−1^	^*^96% at 350 °C ^**^10.6 ×10^−4^ at 350 °C ^**^3.6 ×10^−4^ at 180 °C	Reina et al., 2013a
Au/FeO_x_/CeO_2_- Al_2_O_3_	Al_2_O_3_−73.1 CeO_2_−19.1 Fe_2_O_3_−6.59	1.26	21.0	DAE	IMP on 20 CeO_2_−80 Al_2_O_3_ (Sasol)	0.1 cm^3^; 4.2% CO, 16.2% H_2_O, 79.6% He; GHSV 18 000 h^−1^	^*^40% at 350 ^o^C ^**^9.1 ×10^−4^ at 350 ^o^C ^**^0.5 ×10^−4^ at 180 ° C	Reina et al., 2013b
Au/CeO_2_-Al_2_O_3_	CeO_2_-2.5, 10, 20 on Al_2_O_3_	1, 3, 5	18.0	DP with urea	IMP	0.25 g; 5% CO, 15% H2O, 80% He; feed flow rate 100 ml/min	^*^30% at 330 ^o^C	Gunes and Yildirim, 2010
Au/ZnO-CeO_2_/Al_2_O_3_	Al_2_O_3_−83.9; 83.6 CeO_2_−13.8; 13.4 ZnO−0.4; 1.05	1.87, 1.99	4.0	DAE	IMP on Al_2_O_3_ (Sasol)	0.5 cm^3^; 3.4% CO, 25.0% H_2_O, 71.6% Ar; GHSV 4 000 h^−1^	^**^6.52 ×10^−5^ at 250 ^o^C ^**^7.18 ×10^−5^ at 250 °C	Reina et al., 2014a
Au/Ce_1−x_Cu_x_O_2_/Al_2_O_3_	Al_2_O_3_−80 ÷ 86 CeO_2_−5 ÷ 16 CuO−1.6 ÷ 7.5	1.6÷2.1	n.d.	DAE	Co-IMP on Al_2_O_3_ (Sasol)	0.5 cm^3^; 4.5% CO, 30% H_2_O in N_2_GHSV 4 000 h^−1^	^*^68.2% at 180 °C ^*^96.1% at 300 °C	Reina et al., 2016a
Au/CuO-ZnO-Al_2_O_3_	Al_2_O_3_−37.4÷46.6 CuO−35.6 ÷ 43.6 ZnO−8.7 ÷ 25.8	0.9÷1.2	n.d.	DAE	CP at low saturation	0.5 cm^3^; 3.4% CO, 25.0% H_2_O, 71.6% Ar; GHSV 4 000 h^−1^	80% at 160 °C ^*^Equilibrium conversion at 180 °C	Santos et al., 2017
Au/CuO-ZnO-Al_2_O_3_	Al_2_O_3_−37.4÷46.6 CuO−35.6 ÷ 43.6 ZnO - 8.7 ÷ 25.8	0.9÷1.2	n.d.	DAE	CP at low saturation	0.5 cm^3^; 9% CO, 30% H_2_O, 11% CO_2_, 50% H_2_, GHSV 4 000 h^−1^	^d^ ^**^5.8 ÷ 6.5 ×10^−4^ at 180 °C	Santos et al., 2017
Au/CuO-ZnO-Al_2_O_3_	Cu/ZnO/Al_2_O_3_ hydrotalcites with Cu/Zn = 5.6 and M^2+^/M^3+^ = 1, 2, 3	2.6÷2.8	3.5	DP	CP at low saturation	0.5 cm^3^; 3.4% CO, 25.0% H_2_O, 71.6% Ar; GHSV 4 000 h^−1^	^*^Equilibrium conversion at 170 ° C	Santos et al., 2018
Au/Cu-Mn/ Al_2_O_3_	Al_2_O_3_−78.4 CuO−3.3 ÷13.1 MnO_2_−6.5 ÷ 16.3	2.0	n.d.	DP	IWI on Al_2_O_3_	0.5 cm^3^; 3.4% CO, 25.0% H_2_O, 71.6% Ar; GHSV 4 000 h^−1^	^*^96% at 260 °C	Tabakova et al., 2018

a *Synthesis method of gold-containing catalysts: DAE, direct anion exchange; DP, deposition-precipitation*.

b *Synthesis method of support: IMP, impregnation; IWI, incipient wetness impregnation; CP, coprecipitation*.

c WGS activity of the most active sample in a series:

* CO conversion (%);

** *r (mol_CO_$g_{Au}^{-1}$ s^−1^)*.

d *WGS rate (mol_COconverted_ s^−1^${mol}_{active\;phase}^{-1}\times$10^−4^)—active phase = sum in moles of Au and Cu*.

*n.d, not detectable*.

Analysis of the economic feasibility in case of possible commercial application provokes the important question about the stability and durability of supported gold catalysts. It is known that an important drawback of the gold-based catalysts is related to agglomeration of nanosized gold particles into larger entities, which causes a decrease of catalytic activity (Zhou et al., 2015). However, very recent findings of Behm and co-workers, based on time-resolved operando XAS (XANES/EXAFS) and *in situ* DRIFTS measurements during WGS reaction at 180°C on Au/CeO_2_, allowed one to ignore gold particle agglomeration as a reason for catalyst deactivation (Abdel-Mageed et al., 2017). Accumulation of adsorbed species and blocking of active sites contributed to the deactivation mechanism. This aspect of WGS behavior of the Au/Fe-CeO_2_/Al_2_O_3_ catalyst was examined within 30 h at 250°C (Reina et al., 2013a). A progressively decreased CO conversion has been assigned to selective blocking of active sites for water dissociation due to accumulation of carbonaceous reaction intermediates (most probably formates and/or carboxylates). Additional experiments indicated that this deactivation was fully reversible and the initial activity was recovered after an oxidative treatment, thus implying that the stability could be preserved under suitable reaction conditions. In this context, Deng and Flytzani-Stephanopoulos demonstrated that a small amount of oxygen added to the WGS reaction gas mixture beneficially affected the stability of Au/CeO_2_ catalysts, especially during the shutdown/restart operation (Deng and Flytzani-Stephanopoulos, 2006).

Time-resolved X-ray diffraction and XANES measurements helped to gain further insight into the role of iron to promote WGS activity of Au/ceria catalysts (Reina et al., 2013b). Two types of supports for gold-containing catalysts were prepared aiming to form either a FeO_x_-CeO_2_ solid solution on the surface of γ-alumina or a separate Fe_2_O_3_ phase onto commercial CeO_2_-Al_2_O_3_ (20% CeO_2_-80% Al_2_O_3_). Au/FeO_x_-CeO_2_/Al_2_O_3_ exhibited a significantly higher WGS activity as compared to that of the Au/FeO_x_/CeO_2_-Al_2_O_3_ catalyst. Formation of Ce-Fe solid solutions contributed to higher gold dispersion and enhanced generation of oxygen vacancies, thus favoring water dissociation during WGS reaction. Au/FeO_x_/CeO_2_-Al_2_O_3_ lower activity was associated with co-existence of Fe^3+^ and Fe^2+^ in the magnetite formed during the WGS reaction. However, the reason for the observed catalytic behavior is probably not the presence of a separate magnetite phase, which is well-known as the active working phase of commercial high-temperature WGS catalysts, but suppressed ceria–iron interaction due to employed preparation methods. The use of commercial CeO_2_-Al_2_O_3_ support impedes successful modification of ceria by iron as evidenced by analysis of the reduction behavior. In this connection, some papers were published. Andreeva et al. (2006) have reported an example for the effect of prepared Ce–Al mixed oxide (10 or 20 wt.% Al_2_O_3_) on the WGS activity of gold catalysts. TPR and Raman spectroscopy measurements provided evidences for enhanced formation of oxygen vacancies, but their location deep in the ceria structure hampered diffusion to the surface. Moreover, comparison with Au/ceria catalyst reduction behavior after re-oxidation at room temperature or at 200°C indicated a significantly lower oxygen capacity of Au/CeO_2_-Al_2_O_3_ suggesting straitened re-oxidation of the oxide surface. Vecchietti et al. (2011) have published similar results by comparing WGS performance of gold-based catalysts on CeO_2_, Ga_2_O_3_, and Ce–Ga mixed oxide supports prepared by co-precipitation. CO conversion followed the order: Au/CeO_2_ > Au/Ce80Ga20 >> Au/Ga_2_O_3_. Based on TPR profiles of the supports, the authors concluded that Ce80Ga20 reducibility in comparison with that of ceria was not a proof of improved ability for re-oxidation by water, thus affecting WGS activity. Gunes and Yildirim (2010) have prepared Au/CeO_2_-Al_2_O_3_ catalysts by impregnation of alumina with 2.5, 10, and 20 wt.% ceria followed by homogeneous deposition of gold using urea. The catalysts with 10 and 20 wt.% ceria demonstrated almost the same WGS activity at 330°C that was far below equilibrium. The reason should be a relatively high average size of the gold particles (18 nm measured by XRD). However, a relevant conclusion could not be drawn due to lack of sufficient characterization data including evaluation of reduction behavior.

Further studies focused on the development of highly efficient Au/CeO_2_/Al_2_O_3_ catalysts by evaluation of the promotional effect of Fe, Cu, and Zn (2 wt.% of metal oxide) as ceria dopants on the WGS activity (Reina et al., 2015a). It was found that all dopants contributed to enhanced CO conversion in comparison with undoped gold–ceria–alumina. However, the influence of iron was shown to be the most favorable for the activity. Sample characterization by XRD and Raman spectroscopy in combination with oxygen storage capacity measurements revealed that iron-modified ceria affected simultaneously electronic and structural features in a way to achieve remarkable activity improvement. Additionally, this catalyst composition exhibited a very promising stability during 140 h of continuous operation at 330°C. The redox transfer Fe^+3^ ↔ Fe^2+^ was considered a factor that allows preventing ceria over-reduction, thus avoiding catalyst deactivation.

**Figure 4:**
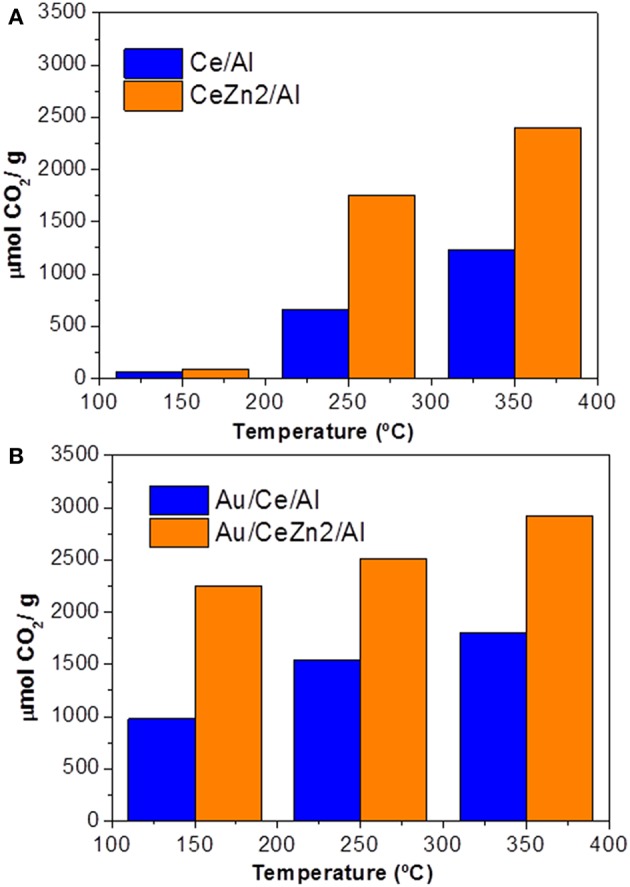
OSCC of the prepared materials: **(A)** supports; **(B)** gold catalysts. Reprinted from Reina et al. (2014a) with permission from John Wiley and Sons.

Parallel to iron activity to promote Au/CeO_2_/Al_2_O_3_ WGS performance, ZnO content (0.40 and 1.05 wt.%) impact has been discussed as ZnO is an efficient structural defect promoter of ceria (Reina et al., 2014a). Catalyst activity measurements clearly manifested the ZnO-promoting effect on ceria in enhancing WGS performance, the catalyst with higher ZnO content being more active. Based on calculations of oxygen storage complete capacity (OSCC) of alumina-supported Ce–Zn formulations that were used to prepare gold-containing catalysts, a further insight into ZnO promotional effect on reducibility was obtained. Zn-promoted supports exhibited higher OSCC values compared to unmodified carriers, indicating a beneficial role of ZnO in oxygen mobility. Again, the gold catalysts showed a much higher OSCC than the corresponding supports, suggesting that gold nanoparticle's ability strongly affects oxygen mobility in the ceria lattice (Figure 4).

The WGS activity of multicomponent Au/Ce_1−x_Cu_x_O_2_/Al_2_O_3_ catalysts of varying CuO amount (1.6, 4.5, and 7.5 wt.%) was significantly affected by the Ce/Cu ratio (Reina et al., 2016a). Both Ce–Cu synergy and strong gold–support interaction governed reduction and catalytic behavior. Noting CO conversion degree, a very relevant comparison between the aforementioned catalyst system and reference WGS catalysts in the literature including commercial low-temperature shift catalyst, all of them tested under the same WGS conditions, confirmed the superiority of the catalyst with the highest CuO content. The study of these new catalytic materials of complex composition has provided very interesting information on how structural and electronic interactions among Au, Cu, and ceria can be carefully tuned to achieve the best catalytic performance. Promising results were obtained for the stability of the most active Au/CeO_2_/Al_2_O_3_ catalysts promoted by CuO and ZnO by analyzing long-term activity tests. The latter included examinations under realistic reformate stream in combination with start-up/shutdown cycling that mimic a real application in fuel processors.

The advantages of the so-called “supported approach” were successfully demonstrated by an in-depth comparison of structure–WGS reactivity relationship of gold deposited on either CuO–CeO_2_ (15 wt.% CuO and 85 wt.% CeO_2_) or alumina-supported mixed CuO–CeO_2_ (25 wt.% in total) (Reina et al., 2016b). Generally, the suitability of supported compositions was evidenced. In particular, a low amount of active phase dispersed on alumina was pointed out to be effective according to specific activity calculations bearing in mind total active phase loading. The results highlighted the importance of high surface-to-volume ratio as a key feature of catalysts with improved catalytic performances. The benefits of this new strategy are clearly stated from the economic and environmental point of view. The results were published in a Special issue of Applied Catalysis B: Environmental entitled: “Forty years of catalysis by ceria: A success story” and revealed the ambitious idea of the authors to develop well-performing catalysts for pure hydrogen production by reducing the amount of ceria, thus saving the world's resources of cerium as one of the important rare earth elements and extending its lifetime “for another 40 years of successful applications in catalysis.”

Favorable properties of transition metal oxides and gold nanoparticles have been combined to prepare gold-based catalysts using alumina-supported Cu–Mn mixed oxides (Tabakova et al., 2018). Sample characterization by several techniques [X-ray diffraction (XRD), High-resolution transmission electron microscopy (HRTEM), Electron paramagnetic resonance (EPR), X-ray photoelectron spectroscopy (XPS), temperature-programmed reduction (TPR)] before and after WGS tests evidenced that the beneficial effect of gold promotion was related to CO activation and enhanced CuO reducibility. Introduced gold contributed to decomposition of Cu_1.5_Mn_1.5_O_4_ spinel and formation of finely divided copper particles during the reaction, avoiding the need of activation pretreatment. It was found that the high activity originated not only from the gold-assisted reduction of CuO but also from the presence of two active metal phases, their dispersion being strongly affected by the composition of the supported Cu-Mn mixed oxides. The role of high interfacial area for stabilizing the gold and copper particles was underlined, thus affecting catalyst stability.

**Figure 5:**
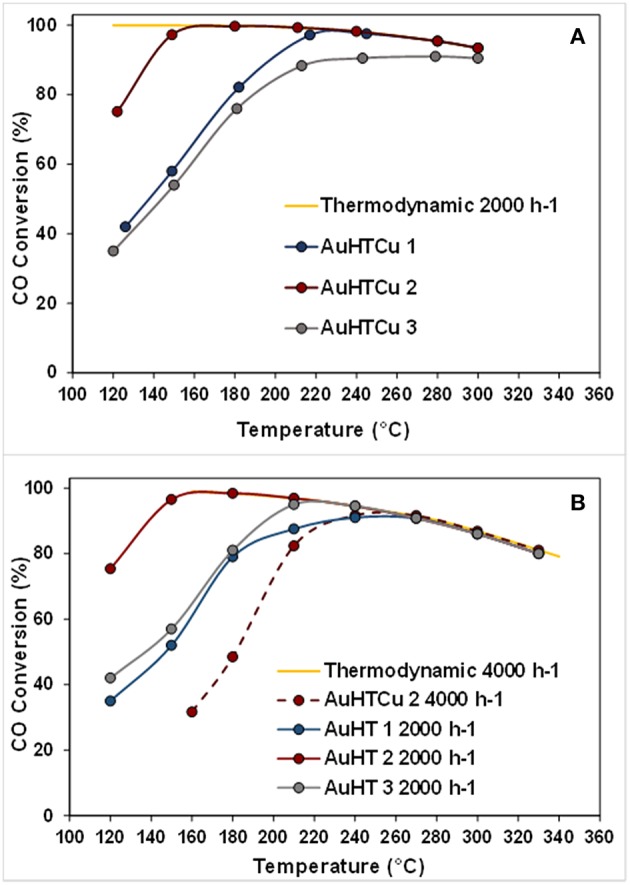
Temperature dependence of CO conversion over the studied gold-based catalysts: **(A)** model WGS mixture−4,5% CO, 30% H_2_O, and N_2_ as balance, GHSV 4000 h^−1^; **(B)** simulated post-reforming mixture−9% CO, 30% H_2_0, 11% CO_2_, and 50% H_2_, GHSV 2000 h^−1^ and 4000 h^−1^(dashed line). Reprinted from Santos et al. (2018). This article is available under the terms of the Creative Commons Attribution License.

Research efforts of Santos et al. (2017, 2018) for exploration of appropriate and economically viable materials resulted in development of advanced catalysts of complex compositions. Considering the advantages and drawbacks of commercial Cu/ZnO/Al_2_O_3_ formulations for large-scale low-temperature WGS, the authors proposed an original new catalytic system based on gold-modified CuO-ZnO-Al_2_O_3_ mixed oxides derived from hydrotalcite precursors. A remarkably high WGS activity of gold-based catalysts in both model and post reforming mixtures (Figure 5) combined with high resistance to deactivation during long-term tests and under simulated start-up/shutdown conditions was accomplished by optimizing the active components ratio and the method of gold deposition (Odriozola et al., 2016). The WGS activity of layered double hydroxides (LDHs) with Ni^2+^/Al^3+^ molar ratios of 1.5, 2.5, and 4.0 was studied, and the more promising samples were promoted by gold (Gabrovska et al., 2019). The layered structure collapsed during conditioning of the catalysts, and formation of a highly dispersed NiO phase (average size of 3.0 nm) was registered in the presence of gold. The role of Au to improve WGS performance was observed with a gold catalyst on LDH with a Ni^2+^/Al^3+^ molar ratio of 2.5, which reached an equilibrium CO conversion of 97.6% at 240°C. It was interesting to note that gold particle size was relatively high—about 15 nm. However, in this case, the larger size was not a catalyst drawback. The ability of very small metallic gold particles (about 1 nm) to dissociate hydrogen even at room temperature has been reported by Boccuzzi et al. (1999). These active hydrogen atoms are spilt over on the support and may cause reduction of the support surface sites. Developing Ni-based catalysts for WGS reaction should always be carefully considered to prevent formation of metallic nickel, which is responsible for methanation—a side reaction that consumes hydrogen and should be avoided during WGS reaction.

Liu et al. (2019) have reported a new route for design of gold-based catalysts and attributed high-performance, satisfactory stability, and recyclability to the influence of electronic metal–support interaction on the electronic structure of metal–support interfacial sites. An in-depth characterization by several *in situ* techniques provided experimental evidences for identification of interfacial active sites, i.e., Au^δ−^−O_*v*_−Ti^3+^. This electron-enriched Au^δ−^ species was related to enhanced CO chemisorption, while O_v_−Ti^3+^ contributed to the dissociation of water. A highest surface concentration of electron-enriched gold species (Au^δ−^) was found by XPS in the most active Au@TiO_2−x_/ZnO sample reduced at 300°C. *In situ* DRIFT spectrum of this sample revealed a strong absorption band at 2067 cm^−t^ assigned to CO adsorbed on negatively charged small gold clusters. The latter occurred because of an electron transfer from the reduced supports to the clusters in agreement with the finding of Boccuzzi et al. (1999) who reported for the first time formation of Au^δ−^ on reduced Au/TiO_2_.

#### Design of Single-Atom Gold Catalysts

**Figure 6:**
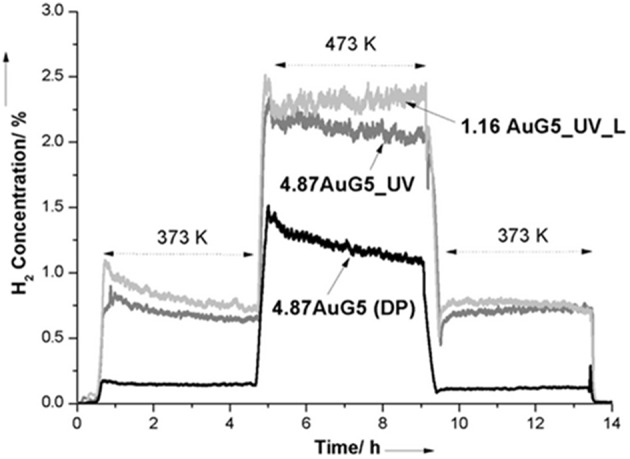
Time-dependent hydrogen production during temperature-programmed surface WGS reaction with 4-h steady state holds (10% CO, 3% H_2_O in He). Reprinted with permission from Yang et al. (2013b), *J. Am. Chem. Soc*. 135, 3768–3771. Copyright (2013) American Chemical Society.

Preparation of atomically dispersed supported metal catalysts could also be considered an attractive approach to developing well-performing and cost-effective catalysts. Single-atom catalysts have been described as a new type of catalytic materials with great potential applications due to the opportunity to maximize metal atom efficiency and minimize costs, using noble metals in particular (Yang et al., 2013a). Flytzani-Stephanopoulos and co-workers have successfully applied this concept to gold catalysts for the WGS reaction. In 2003, they presented the first evidence for stabilized gold in ceria in the form of single-site Au-O_x_- species. This non-metallic species embedded in ceria was considered an active site for the WGS reaction (Fu et al., 2003). To increase abundance of the atomically dispersed gold species, different methods were adopted depending on support type and structure. Cyanide leaching was carried out for total removal of weakly bonded gold nanoparticles and clusters (over 90% of gold loading) from ceria and iron oxide. It is known that nanosized metallic gold particles are able to agglomerate during the reaction at elevated temperatures, thus negatively affecting catalyst stability. In contrast, low-content Au–ceria catalysts exhibited good stability in various gas compositions and temperatures and during simulated shutdown operations (Fu et al., 2005). In the case of titania-supported gold catalysts, it was demonstrated that UV irradiation of uncalcined Au/TiO_2_ effectively contributed to activation of surface vacancies on the titania and to binding the atomically dispersed gold species on the titania surface, thus enhancing significantly WGS activity (Figure 6) (Yang et al., 2013b). The same group has developed a new method allowing stabilization of atomic gold species on any support including irreducible oxides, such as silica, alumina, and zeolites (Yang et al., 2014). These support surfaces are not able to provide enough –O ligands that should coordinate with a gold atom and stabilize it. Alkali ions (Na or K) activated and stabilized atomic gold through –O–Na(K) bonds on inert zeolite and mesoporous MCM-41 in the form of AuO_y_(OH)_z_(Na or K)_x_ clusters that exhibited a high activity in the WGS reaction due to weak CO adsorption on these structures and facilitated water dissociation to –OH and –H. It was interesting to find the same intrinsic activity of the single-site gold species on reducible CeO_2_, Fe_2_O_3_, and TiO_2_ as on irreducible supports, suggesting the existence of gold active site of similar structure. Flytzani-Stephanopoulos has summarized the knowledge gathered over the last years about the origin of the active gold sites for WGS reaction in two recent publications (Flytzani-Stephanopoulos, 2014; Yang and Flytzani-Stephanopoulos, 2017). More details are reported about the methods assuring preparation of atomic dispersions of gold on different supports. The findings can serve as guidelines for design of new gold catalysts with superior gold site efficiency and stable activity in realistic WGS operating conditions.

**Figure 7:**
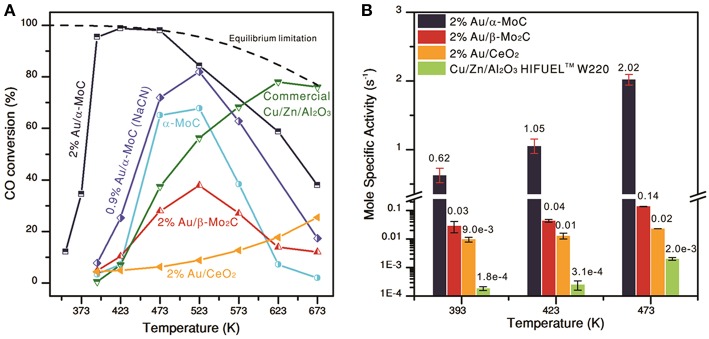
**(A)** Temperature dependence of CO conversion on different catalysts with model WGS mixture: 10.5% CO, 21% H_2_O, 20% N_2_ in Ar and GHSV 180,000h^−1^; **(B)** specific activity of different catalysts measured at CO conversion below 15% in simulated post-reforming mixture 11% CO, 26% H_2_O, 26% H_2_, 7% CO_2_, and 30% N_2_. From Yao et al. (2017), *Science* 357, 389–393. Reprinted with permission from AAAS.

Transition metal carbides were also used as support materials due to their relatively low cost and interesting physical and chemical properties (Posada-Pérez et al., 2017). WGS performance of atomic-layered gold clusters (2 wt.% Au) supported on α-MoC was studied (Yao et al., 2017). High-resolution STEM Z-contrast images indicated that epitaxial Au layered clusters decorating the α-MoC support (with an average diameter of 1 to 2 nm and a thickness of 2 to 4 atomic layers) coexisted with atomically dispersed gold. Comparison between WGS activity of this sample with that of α-MoC, 2%Au/β-Mo_2_C, 2%Au/SiO_2_, and 2%Au/CeO_2_ showed a very high CO conversion over 2%Au/α-MoC (> 95% at 120°C and 98% at 150°C) (Figure 7). However, a decrease in Au content normalized WGS activity after leaching with NaCN (Au content about 0.9 wt.%) revealed that high WGS activity at a low temperature should be attributed mainly to gold-layered clusters epitaxially grown on the α-MoC.

#### Preparation of Efficient Supports for Gold WGS Catalysts

Recently, hydrogen-etching technology was applied as a successful approach to stabilize oxygen vacancies on the surface of TiO_2_ (Li et al., 2018). According to the synthesis procedure, almost white anatase was prepared after calcination in air, while thermal treatment at 550°C for 4 h under high-purity H_2_ atmosphere caused its transformation into black TiO_2−x_ support. Gold deposited on this black anatase exhibited a higher and more stable WGS activity than that of gold supported on traditional white titania, as shown by comparing temperature dependences of CO conversion, TOFs, and CO conversion during a 30-h stability test at 600°C. A detailed structural study based on several techniques (XRD, HRTEM, EPR, Raman and XP spectroscopy, H_2_-TPR) revealed that the hydrogen-etching method contributed to abundance of stable surface oxygen vacancies, which facilitated availability of more metallic gold species at the surface and a higher WGS activity. Some of the authors of this paper also applied hydrogen-etching technology to prepare blue-black TiO_2−x_ nanoribbons and arrived at very similar conclusions for the effect of reduction treatment to increase the number of oxygen vacancies and to improve water activation, thus enhancing WGS activity (Song et al., 2018).

**Figure 8:**
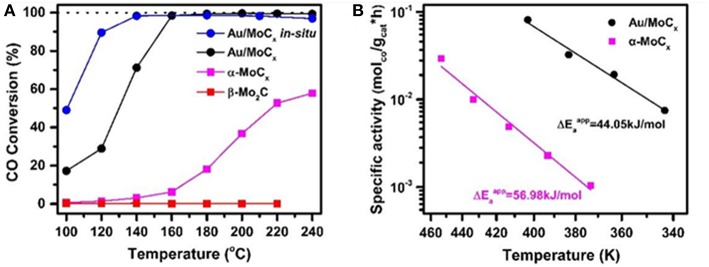
**(A)** Temperature dependence of CO conversion over Au/MoC_*x*_, α-MoC_1−*x*_, β-Mo_2_*C*, and *in-situ* synthesized Au/MoC_*x*_ samples; **(B)** Arrhenius plots of LT-WGSR rates of Au/MoC_*x*_ and α− MoC_1−*x*_ samples at CO conversion below 15%. Reproduced with permission from Dong et al. (2018), *J. Am. Chem. Soc*. 140 (42), 13808–13816. Copyright (2018) American Chemical Society.

In an attempt to elaborate the SMSI concept in the case of metal/carbide interface, one-step carbonization process has been applied for preparation of gold catalysts using molybdenum carbide (MoC_x_) (Dong et al., 2018). Formation of highly dispersed Au layers over MoC_x_ was assigned to a strong interfacial charge transfer between the gold and the Mo carbides that resulted in a very high WGS activity at a low temperature (100% CO conversion at 140°C) (Figure 8). The registered degree of CO conversion was comparable to the result on Au/α-MoC reported by Yao et al. (2017). The applicability of this type of gold catalysts was demonstrated based on stability tests that indicated complete recovery of the performance after oxidative activation.

### Structured (Monolithic) Catalysts for WGS Reaction

The economic viability of high-purity hydrogen production for small-scale applications is strongly dependent not only on developing efficient catalytic materials but also on a suitable reactor design. One of the main problems on using pelletized powder catalysts is pressure drop during the catalytic reaction, which reduces flow rates of the reactant gases. Moreover, these catalysts suffer from breakage due to stresses originated from frequent start-up/shutdown operations in the mobile fuel cell systems. Structured catalysts, also known as monoliths, offer significant advantages in comparison with particulate catalyst beds. They could overcome the problems arising from WGS peculiarities due to their high mechanical and chemical durability, low pressure drop, rapid response to transient operation, and smaller sizes (Farrauto et al., 2007; Ivanova et al., 2013). Teo et al. (2011) have performed a systematic study on synthesis and application of supported gold monolithic catalysts in the WGS reaction and achieved very promising results by comparing commercial high- and low-temperature WGS catalysts. Sonochemical, co-precipitation, and deposition–precipitation methods of preparation of Au/Fe_2_O_3_ powder catalyst and various treatments during the wash-coating process (solvents, binders, and different pH values) have been evaluated with respect to WGS activity. A favorable use of α-Fe_2_O_3_ nanofibers instead of powder Fe_2_O_3_ was demonstrated. Catalyst stability was significantly improved by addition of ZrO_2_ as shown after about 400-h tests in a realistic reformate gas mixture. The benefits of Au/ceria series as active WGS catalysts were successfully combined with those of cordierite monoliths (Özyönüm and Yildirim, 2016). Moreover, commercial cordierite substrates with thin walls and 400 cells per square inch were used, thus further manifesting the advantage of microchannel reactors as an approach to miniaturize reactors for fuel cell applications. Rhenium added as a second metal component improved the catalytic activity under both idealistic and realistic feed conditions over the full temperature range.

Catalysts for WGS reaction prepared on metallic monoliths are another attractive opportunity for realization of this process in hydrogen fuel processors. Metallic monoliths have important advantages over ceramic materials, one of the most favorable being a greater mechanical strength and a higher thermal conductivity (Sanz et al., 2013). González-Castaño et al. (2016) have compared the WGS performance of Au and Pt supported on Fe-doped ceria in the form of powder or structured catalysts. Both catalysts supported on metallic monoliths exhibited a lower activity with respect to their powder analogs, but the platinum-based samples were better than the gold entities. However, small amounts of oxygen in the WGS feed mixture significantly improved the activity of the gold-based monolith, focusing on the role of oxygen in the so-called “O_2_-assisted WGS” for accelerating ceria re-oxidation by water and facilitating decomposition of carbonaceous intermediate species (Deng and Flytzani-Stephanopoulos, 2006; Kugai et al., 2011; Reina et al., 2013a).

## Latest Mechanistic Aspects of the WGS Reaction Over GOLD-Based Catalysts

Detailed review of the nature of active sites in Au-based catalysts under WGS reaction conditions and operating reaction mechanism is out of the aim and scope of this work. Mechanistic aspects of the WGS reaction over gold-based catalysts have been critically reviewed in many of the abovementioned publications (Burch, 2006; Ratnasamy and Wagner, 2009; Andreeva et al., 2013; Liu, 2013; Tao and Ma, 2013; Ramirez Reina et al., 2014; Reddy and Smirniotis, 2015; Carter and Hutchings, 2018). Therefore, some very recent results are only noted. Two reaction mechanisms have mainly been considered in the literature: a regenerative redox mechanism and an associative mechanism. Burch (2006) has proposed a universal WGS mechanism. The majority opinion is that the dominant mechanism depends on the reaction conditions, in particular temperature and H_2_O/CO_2_ ratio. The redox mechanism would be expected to prevail at higher temperatures. At low temperatures and depending on water concentration in the reaction mixture, carbonate or formate decomposition steps would be more important (Andreeva et al., 2013). Despite intensive research and use of advanced characterization methods, debates concerning the mechanism for gold-catalyzed WGS reaction are still open. As a confirmation, some latest contributions to this topic, based on DFT calculations, have reported quite different findings. By combination of DFT calculations and experimental study of Au/TiO_2_, Au/Y_2_O_3_, and Au/TiO_2_-Y_2_O_3_, Plata et al. (2018) have suggested carboxyl formation as the rate-limiting step of the WGS reaction. The authors stressed on the role of homogeneously dispersed vacancies to act both as nucleation centers for small gold particles and as active sites for water dissociation. Song and Hensen (2014) have shown that WGS reaction over two different models, i.e., isolated and clustered Au atoms on CeO_2_(110), proceeds preferentially by a carboxyl mechanism. Formation of oxygen vacancies and COOH species from CO and ceria surface OH groups were identified as rate-limiting steps. In contrast, Sun et al. (2017) have found that the formation of COOH species is very difficult to occur during WGS reaction on Au/TiO_2_ catalysts. Analysis of DFT calculations and micro-kinetic studies suggested the redox mechanism as the primary reaction pathway. A DFT study of the WGS reaction mechanism over Au_10_, Au_13_, and Au_20_ clusters indicated that a carboxyl mechanism operated over Au_10_ and Au_20_ clusters, while a redox reaction mechanism functioned over the most active Au_13_ cluster (Zhang et al., 2017). Upon targeting elucidation of the role of support oxygen and subsurface dynamics, a redox mechanism for the WGS reaction over Au/ceria catalysts has been proposed based on DFT+U calculations and *in situ* and *in operando* Raman spectroscopic study (Schilling and Hess, 2019).

Studies of model catalysts contribute significantly to understanding different aspects of the WGS reaction mechanism. Rodriguez et al. (2017) added new insights into correlations among structural, electronic, and catalytic properties of gold–metal oxide interfaces by focusing on the importance of strong metal–support interactions. An enhanced interaction between Au nanoparticles and CeO_2_ after modification with Ti was verified in theory and reported as an approach to improving sintering resistance (Zhu et al., 2018).

**Figure 9:**
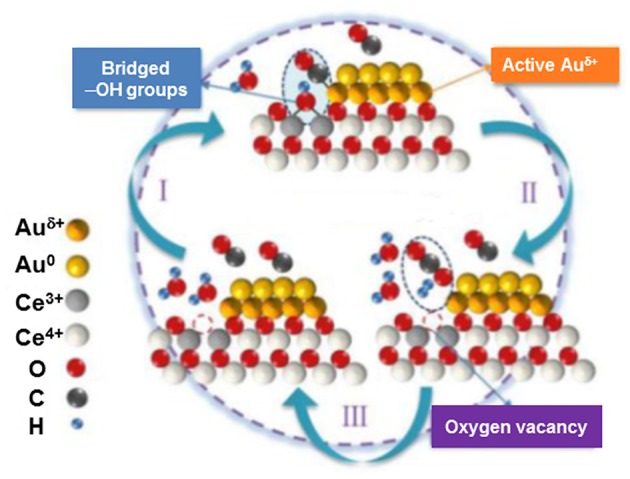
Graphic representation of the WGS reaction mechanism on the interface of Au/CeO_2_ catalysts. Reproduced with permission from Fu et al. (2019), *J. Am. Chem. Soc*. 141 (11) 4613–4623. Copyright (2019) American Chemical Society.

Very recently, a detailed study has been carried out to identify active surface species and give new insights into the WGS reaction mechanism over Au/CeO_2_ (Fu et al., 2019). Two catalysts were precisely designed to contain gold clusters (<2 nm) and gold particles (3–4 nm) on ceria nanorods in order to have reliable comparison of gold–support interfacial structures. By using comprehensive *in situ* characterization techniques, a crucial role of the interaction between bridged surface OH groups and CO adsorbed on interfacial gold atoms has been underlined. Superior reactivity of Au clusters/ceria was attributed to abundance of interfacial sites and particularly to the contribution of CO-Au^δ+^ species (Figure 9). This conclusion corroborates earlier findings of Tabakova et al. (2010) who have highlighted a higher WGS activity because of highly dispersed gold clusters in contact with oxygen vacancies on ceria surface.

## Well-Performing Gold Catalysts for Additional Hydrogen Clean-Up by PROX

Efficient upgrading of hydrogen purity for fuel cell applications cannot be achieved by WGS reaction because it is an equilibrium-limited reaction, i.e., a lower temperature favors a higher CO removal. A mandatory clean-up stage is required to ensure CO removal (tolerable level below 10 ppm) in order to avoid poisoning of sensitive Pt anode in fuel cells such as PEM types. Many authors consider the CO PROX as “the simplest and most cost-effective” method for elimination of CO from the hydrogen-rich gas stream. Development of a suitable catalyst for PROX is challenging because it should demonstrate high CO activity and high selectivity to avoid undesired hydrogen oxidation at a low temperature (80–120°C). An important requirement that should also be fulfilled is tolerance toward the presence of CO_2_ and H_2_O in the feed. Mishra and Prasad (2011) have summarized the promising catalysts for PROX into three groups: (i) metal oxide-supported noble metal catalysts (Pt, Pd, Ir, Ru, or Rh), (ii) gold-based catalysts, and (iii) transition metals (Co, Cu, Fe, Ni, Zn, Mn) supported on metal oxides. Avgouropoulos et al. (2002) presented the first comparative study of Pt/γ-Al_2_O_3_, Au/α-Fe_2_O_3_, and CuO–CeO_2_ catalysts for PROX. Au/α-Fe_2_O_3_ demonstrated a superior selective CO oxidation at the operating temperature of PEM fuel cells (80–120°C) regardless of reactant feed composition, i.e., in the presence of CO_2_ or both CO_2_ and H_2_O. Bion et al. (2008) analyzed advantages and drawbacks of noble metals (Pt, Ru, Rh, Pd), gold, and transition metal oxide catalysts in PROX and concluded that, generally, Au-based catalysts are more active and selective in comparison with Pt catalysts in a large temperature interval. Alumina-supported Pt catalysts are most commonly applied for PROX reaction. Although these catalysts exhibit a high CO oxidation activity, they need high operation temperatures (150–200°C) and full control of the reaction conditions. A serious drawback is the effect of space velocity, because low values favor the reverse WGS reaction and formation of CO. In contrast, gold-based catalysts manifest high performance for CO oxidation at lower temperatures. A further advantage is that the rate of CO oxidation over supported Au catalysts exceeds that of H_2_ oxidation (Haruta et al., 1993; Grisel et al., 2002). Kandoi et al. (2004) have used DFT calculations and micro-kinetic modeling to show that the energy barrier for CO oxidation on Au(111) facets is lower than that for H_2_ oxidation, thus explaining the higher selectivity of gold-based catalysts in comparison with platinum-based catalysts in PROX at low temperatures.

Recently, Lakshmanan et al. (2014) summarized progress in developing gold catalysts for PROX reaction. The nature of support is one of the most important factors affecting gold-based catalyst efficiency. Depending on activity in H_2_ and CO oxidation, this was highlighted by classification of carrier materials into three groups. Support effects on PROX activity of gold catalysts have been studied by Liotta et al. (2010) by emphasizing a favorable role of reducible oxides (Co_3_O_4_-CeO_2_, CeO_2_, and Co_3_O_4_) for stronger metal–support interaction and enhanced oxygen vacancies formation in comparison with gold supported on non-reducible alumina. A reliable assessment of support effects in the PROX reaction was performed by Ivanova et al. by applying “on-surface bottom-up” approach to prepare gold catalysts of almost identical gold particle size, shape, and oxidation state on supports being regarded as active (TiO_2_, ZrO_2_), inert (Al_2_O_3_), and reducible (CeO_2_) (Ivanova et al., 2010). The findings underlined gold availability in a fully reduced state and the role of water formation to increase the relative number of active sites as important factors to achieve high CO oxidation rates at low temperatures. On analyzing support effects, Beck et al. (2018) have shown that gold catalysts supported on either Al_2_O_3_ or ZnO demonstrated very low CO conversions irrespective of gold particle size, while the use of ceria favored a good PROX performance. Chen and Sasirekha (2014) presented a critical review and their viewpoint of PROX over supported gold catalysts with particular focus on various methods for preparation of highly dispersed gold particles, the role of the support, and the effect of support modification. A beneficial promotional role of various metal oxides defined as “active,” such as MnO_x_, CeO_2_, CuO_x_, Fe_2_O_3_, and CoO_x_, and “inactive,” like ZnO and MgO, on the catalytic performance for PROX over Au/TiO_2_ has been reported. Ma et al. (2015) have also reviewed the progress in developing new gold catalysts for PROX, focusing on materials of complex metal–support interfaces. Application of various modified metal oxides or mixed metal oxides for preparation of gold catalysts was reported together with studies of bimetallic nanoparticles on the same supports. By assessing current state of the knowledge in the field, the authors share their critical remarks about the lack of fundamental results on the nature of active sites and reaction mechanism and give some perspectives on future research.

### Alumina or Modified Alumina Supports for Gold-Containing Catalysts

Following one of the aims of this review to discuss appropriate and economically viable gold-based catalysts, recent investigations of alumina-supported gold catalysts for PROX will be analyzed. Alumina is one of the most commonly applied commercial carriers in heterogeneous catalysis due to its high surface area, thermal stability, and mechanical strength. Overview of some characteristics of selected gold-based catalysts using alumina or modified alumina supports and their PROX performance is reported in Table 2. Although alumina is a non-reducible oxide and Au/Al_2_O_3_ catalysts exhibit a poor CO oxidation activity, Quinet et al. (2008) have shown that, at low temperatures, small amounts of hydrogen in the reactant mixture enhance CO oxidation rate, thus revealing a promising performance in PROX. The promotional effect of hydrogen was related to stabilization of highly active –OOH species on the surface of gold particles that is able to interact efficiently with CO to form CO_2_. Saavedra et al. (2016) have reported a beneficial function of one to two monolayers of water as CO oxidation co-catalyst during PROX over commercial Au/Al_2_O_3_ catalyst. Carefully tuned water content in the reaction mixture and space velocity significantly affected activity and selectivity to achieve target CO concentration values below 10 ppm and selectivity of 80–90%. The presence of monolayer water also favored the stability; namely, no deactivation within 10 h occurred because of the role of water to avoid active site blocking by reaction intermediates. A very recent study of hydrogen oxidation over Au/TiO_2_ and Au/Al_2_O_3_ catalysts confirmed water function in promoting CO oxidation and inhibiting undesirable H_2_ oxidation, providing surprising kinetic and DFT-based findings for heterolytic H_2_ activation at the metal–support interface (Whittaker et al., 2018).

**Table 2:** Overview of some characteristics of selected gold-based catalysts using alumina or modified alumina supports and their PROX performance.

**Catalyst**	**Composition wt. %**	**Gold content wt.%**	**Gold size nm**	**Synthesis method^**a**^**	**Synthesis method^**b**^**	**Reaction conditions**	CO conversion^c^^*^CO_**2**_ selectivity	**References**
Au/Al_2_O_3_	Al_2_O_3_ −99. 08	0.92	5.8	DAE	Al_2_O_3_ (Axens)	27 mg; 2% CO, 2% O_2_, 48% H_2_ in He; GHSV ~ 2 100 h^−1^	~ 65% at 110 °C	Quinet et al., 2008
Au/Al_2_O_3_ commercial AUROlite™	Al_2_O_3_−98	1.0	2-3	n.r.	n.r.	1% CO, 1.4% O_2_, 60% H_2_, balance He, 1-2 monolayed water SV = 1.4-28 L g^−1^ min^−1^	>10 ppm at 80 °C ^*^80-90%	Saavedra et al., 2016
Au/MgO/Al_2_O_3_	atomic ratio Au:Mg = 1:5	5.0	2.2	DPU	IMP γ-Al_2_O_3_ (Engelhard)	H_2_:CO:O_2_ = 4:2:1, totally 4 vol% in He GHSV 2500 h^−1^	59% at 25 °C/ ^*^about 100% 33% +50 μl H_2_O	Grisel and Nieuwenhuys, 2001
Au/MnO_x_/Al_2_O_3_	atomic ratio Au:Mn = 1:5	4.8	9.2	DPU	IMP γ-Al_2_O_3_ (Engelhard)	H_2_:CO:O_2_ = 4:2:1, totally 4 vol% in He GHSV 2500 h^−1^	49% at 25 °C/ ^*^about 100% 24% +50 μl H_2_O	Grisel and Nieuwenhuys, 2001
Au/MgO/MnO/Al_2_O_3_	atomic ratio Au:Mn:Mg = 1:5:5	4.9	2.7	DPU	HDP γ-Al_2_O_3_ (Engelhard)	H_2_:CO:O_2_ = 4:2:1, totally 4 vol% in He GHSV 2500 h^−1^	100% at 25 °C/ ^*^about 100% 100% +50 μl H_2_O	Grisel and Nieuwenhuys, 2001
Au/MnO_2_-Al_2_O_3_	MnO_2_ - 18.1	3.1	~ 5	DP	Redox Al_2_O_3_ micro-sphere	0.1 g, 1% CO, 1% O_2_, 40% H_2_, N_2_ as balance, flow rate 67 mL/min	100% at 80 °C/ ^*^80%	Miao et al., 2015
Au/MO_x_/Al_2_O_3_M = La, Ce,	CeO_2_−5.29 La_2_O_3_−4.42	0.25 0.30	1.5 2.9	DPU	IMP Al_2_O_3_ (Alfa Aesar)	0.1 g; 1% CO, 1% O_2_, 50% H_2_, He as balance, flow rate 100 mL/min	91.4% at 60 °C ^*^no data 97.6% at 60 °C/ ^*^50-80% at 60 °C	Lakshmanan et al., 2016
Au/MgO/Al_2_O_3_	MgO−4.18	0.31	2.1	DPU	IWI Al_2_O_3_ (Alfa Aesar)	0.1 g; 1% CO, 1% O_2_, 50% H_2_ He as balance, flow rate 100 mL/min	91.5% at 95 °C/ ^*^no data	Lakshmanan et al., 2016
Au/La_2_O_3_/Al_2_O_3_	La_2_O_3_−3.8	0.3	4.2	DPU	IMP Al_2_O_3_ (Alfa Aesar)	0.1 g; 1% CO, 1% O_2_, 50% H_2_, 48% He, flow rate 100 mL/min	92% at 90 °C/ ^*^48.2% with CO_2_ and H_2_O 93% at 97 °C/ ^*^49%	Lakshmanan and Park, 2018
Au/La_2_O_3_/Al_2_O_3_	La−13.2	0.82	1.8	adsorption	IWI γ-Al_2_O_3_	0.05 g; 1% CO, 1% O_2_, 40% H_2_, He as balance, flow rate 50 mL/min	100% at 50-70 °C/ ^*^70-50% at 50-70 °C	Lin et al., 2014
Au/CeO_2_-Al_2_O_3_	CeO_2_−15.9	0.9	1÷2	HAuCl_4_ reduction by THPS	one pot method	1.25% CO, 1.25% O_2_, 50% H_2_ He as balance, W/F = 0.18 g s cm^−3^	100% at 65 °C/ ^*^61%	Storaro et al., 2010
Au/ mesoporousCeO_2_-Al_2_O_3_	CeO_2_−6, 11, 20.8, 29.3, 35.4	0.83÷1	~ 2	IMP	EISA	2 % CO, 2% O_2_, 70% H_2_, 26% He, flow rate 100 mL/min	91-100% at 60 °C/ ^*^49-54%	Fonseca et al., 2012
Au/CeO_2_-Al_2_O_3_	CeO_2_ - 8	0.9	2.6	DP	IMP Al_2_O_3_ HT	1% CO, 1% O_2_, 40% H_2_, N_2_ as balance, flow rate 67 mL/min	97% at 80 - 150 °C/ ^*^~ 50%	Miao et al., 2016
Au/CeO_2_-MO_x_/Al_2_O_3_ M = La, Ni, Cu, Fe, Cr, Y	CeO_2_−13.3 ÷ 16.5 MO_x_ −1.24 ÷ 3.07	1.6÷2.2	<5	DAE	IMP γ-Al_2_O_3_ (Sasol)	0.1 g; 1% CO, 1.5% O_2_, 50% H_2_, N_2_ as balance flow rate 100 mL/min	M = Fe: 90% at 55-80 °C/ ^*^~40-50 M = Cu 90% at 110 °C/ ^*^50%	Reina et al., 2015b
Au/CeO_2_-CuO/Al_2_O_3_	Al_2_O_3_−80.6 CeO_2_−15.8 CuO−1.8	1.75	<5	DAE	IMP γ-Al_2_O_3_ (Sasol)	0.1 g; 1% CO, 1.5% O_2_, 10% H_2_O, 10% CO_2_, 50% H_2_, N_2_ as balance	95% at 110 °C/ ^*^55%	Reina et al., 2015b
Au/MO_x_/Al_2_O_3_ M = Ce, Co	CeO_2_−13.7 ÷ 15.3 Co_3_O_4_−0.4 ÷ 2.8	1.7÷2.2	2÷5	DAE	IMP γ-Al_2_O_3_ (Sasol)	0.1 g; 1% CO, 1.5% O_2_, 10% H_2_O, 10% CO_2_, 50% H_2_, N_2_ as balance flow rate 100 mL/min	70% at 130 °C/ ^*^25%	Reina et al., 2015c
Au-Cu/Fe or La/ Al_2_O_3_	Fe + La−2 Fe : La = 1:1 Cu−0.24, 0.51, 1.04, 1.55	1.2÷1.3	2.0	MDP	IMP γ-Al_2_O_3_ beads	0.2 g; 1% CO, 1% O_2_, 50% H_2_, 48% N_2_, flow rate 100 mL/min	100% at 30-100 °C/ ^*^70% at 60 °C	Sun et al., 2016
Au/Y_2_O_3_-CeO_2_/γ-Al_2_O_3_	CeO_2_−10, 20, 30 Y_2_O_3_−1% in respect to CeO_2_	3	1.9÷2.7	DP	Consecutive IMP γ-Al_2_O_3_ (Sasol)	0.05 g; 1% CO, 1% O_2_, 60% H_2_, He as balance, WHSV 60 000 mL g^−1^ h^−1^	70% at 80 °C/ about 40% +10% CO_2_+10% H_2_O 55% at 100°C/ ^*^30%	Ilieva et al., 2019

a *Synthesis method of gold-containing catalysts: DAE, direct anion exchange; DP, deposition-precipitation; DPU, deposition-precipitation with urea; MDP, modified deposition-precipitation*.

b *Synthesis method of support: IMP, impregnation; IWI, incipient wetness impregnation; CP, coprecipitation; HDP, homogeneous*.

*deposition-precipitation of Mn on calcined MgO/Al_2_O_3_; EISA, evaporation induced self assembly route; Al_2_O_3_ HT, hydrothermally prepared*.

c CO conversion/selectivity of the most active sample: CO conversion (%);

* *Selectivity (%)*.

*n.r, not reported*.

Grisel and Nieuwenhuys (2001) have also considered the role of surface OH groups on the gold-catalyzed PROX. They performed one of the first studies to demonstrate Au/Al_2_O_3_ performance in PROX. Alumina modified by MgO or MnO_x_, or both metal oxides, affected activity and selectivity in CO oxidation to CO_2_ in a positive way. Introduced MgO contributed to formation of gold particles of smaller size, while MnO_x_ improved CO oxidation activity by oxygen activation. Modified alumina microspheres by MnO_2_ favored CO oxidation over gold catalyst (size distribution from 2.5 to 7.5 nm) (Miao et al., 2015). Carbon monoxide was completely oxidized over a 3 wt.% Au catalyst in a temperature range of 80–120°C with 80% selectivity at 80°C. A negative effect on PROX activity in the presence of CO_2_ was attributed to an increased mean size of MnO_2_ after reaction and formation of manganese carbonate in agreement with XRD pattern.

**Figure 10:**
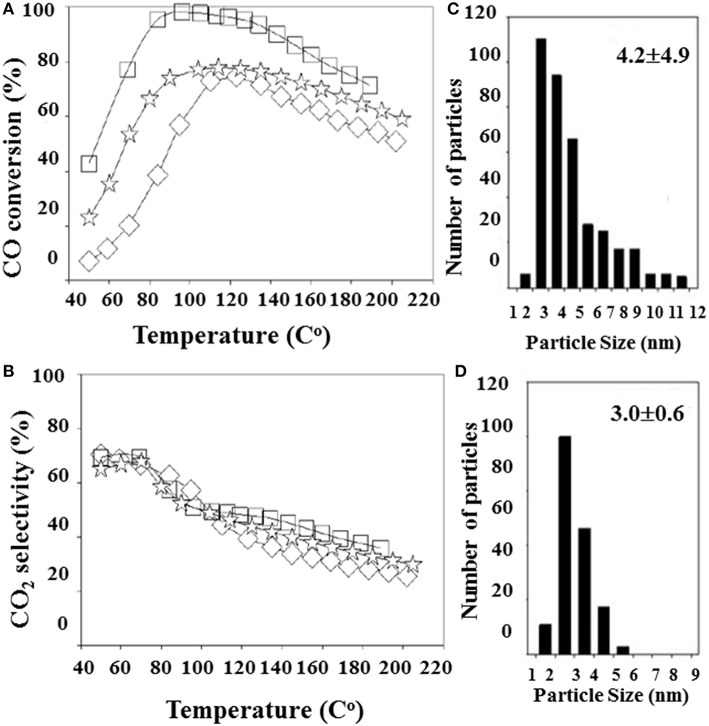
Effect of the reduction method on PROX activity **(A)** and selectivity **(B)** of Au/La_2_O_3_/Al_2_O_3_: (□)- reduction with NaBH_4_/Au molar ratio of 35 (S35), (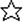) reduction with NaBH_4_/Au molar ratio of 115, and (♢) reduction by glycerol (G). Gold particles size distributions of Au/La_2_O_3_/Al_2_O_3_ samples S35 **(C)** and G **(D)**. Reproduced from (Lakshmanan and Park, 2018). This is an open access article distributed under the Creative Commons Attribution License.

Lakshmanan et al. (2016) have studied the effect of various promoters (La_2_O_3_, CeO_2_, and MgO) on Au/Al_2_O_3_ performance for PROX. In contrast to the aforementioned promotional role of MgO on gold dispersion and PROX activity, Au/MgO/Al_2_O_3_ was at least an active catalyst in this case, probably because of accelerated hydrogen oxidation below 100°C (Grisel and Nieuwenhuys, 2001). The catalytic activity of the best-performing Au/La_2_O_3_/Al_2_O_3_ catalyst was tested after different reduction pretreatments, implying that abundance of negatively charged gold species is beneficial for CO oxidation. A detailed study has been carried out to clarify the reduction treatment effect on Au/La_2_O_3_/Al_2_O_3_ catalytic behavior in PROX (Lakshmanan and Park, 2018). Variation of the CO conversion was related to differences in average gold particle size and oxidation state of the gold species due to different reduction methods (Figure 10). The best performance was demonstrated by a catalyst of 4-nm average gold particle size that was not the smallest size. This finding revealed that decreasing the gold particle size is not the only reason to increase the rate of CO conversion but also undesirable hydrogen oxidation could be affected and a proper modification of alumina could solve the problem with selectivity (Lakshmanan et al., 2016). Modification of commercial γ-Al_2_O_3_ by La (13.2 wt.%) was a successful example in this direction (Lin et al., 2014). An efficient gold catalyst (0.82 wt.% Au) showing a high and stable activity at a temperature window of 50–100°C in the presence of CO_2_ and H_2_O has been developed. It was suggested that the formation of LaAlO_3_ enhanced competitive CO oxidation by decreasing the intrinsic activity for H_2_ oxidation.

Ceria, due to its redox behavior and unique catalytic properties, also became a very interesting support for gold-containing catalysts for PROX (Andreeva et al., 2013; Lakshmanan et al., 2014; Centeno et al., 2016). Its wide application has been dictated by high oxygen storage capacity resulting from oxygen mobility and formation of oxygen vacancies (Trovarelli, 2002; Vindigni et al., 2011). The use of CeO_2_-Al_2_O_3_ mixed oxides as a support for gold catalysts for PROX has been reported in several papers. Storaro et al. have prepared mesoporous alumina–ceria (20 wt.% CeO_2_) of high surface area and uniform pore size distribution by a one-pot procedure and dispersed gold sols on it. The latter were obtained upon reduction of HAuCl_4_ by either bis[tetrakis-(hydroxymethyl)phosphonium sulfate] (THPS) or NaBH_4_ (Storaro et al., 2010). It has been observed that THPS can reduce and stabilize highly dispersed gold particles. Being in close contact with ceria, these particles are uniformly distributed on the pore walls of Ce–Al mixed oxide and contribute to high activity, selectivity, and stability in the presence of CO_2_ and water. Mixed Al–Ce oxides with Ce/Ce+Al ratios of 0, 2, 5, 10, 15, and 20 mol.% were prepared by sol–gel method and then gold was supported by impregnation in organic medium (Fonseca et al., 2012). Al–Ce mixed oxide supported gold catalysts of 5 and 10 mol.% ceria content exhibited the highest CO oxidation activity at a temperature of 60°C with good selectivity (50–60%). A decreased CO conversion over the catalyst of highest ceria content has been attributed to increased basicity of the mixed oxide and facilitated formation of carbonate species on the oxide surface in agreement with findings of Schubert et al. (2004) about the detrimental role of surface carbonaceous species on CO-PROX activity. This suggestion was confirmed on comparing the effect of adding CO_2_ to the reaction mixture on Au/CeO_2_ and Au/Al–Ce (10 mol.% CeO_2_) performance, where a significant deactivation of gold on ceria was observed.

Lab-made alumina was prepared by hydrothermal treatment and modified by ceria (about 8 wt.%) using the impregnation method (Miao et al., 2016). Gold or Au–Cu catalysts were synthesized using an Al–Ce support. Monometallic and bimetallic catalysts manifested superior catalytic behavior with CO conversion >97% and CO_2_ selectivity >50% at a temperature interval of 80–150°C; however, no effect of adding CO_2_ and water was reported. The improved performance of Au or Au-Cu catalysts on Al–Ce support was explained by the role of ceria in assisting anchoring of small metallic particles (1.5–4.0 nm) on the support and to provide reactive oxygen species. Here, on analyzing the favorable features of ceria, it is reasonable to note the preparation of ceria-supported gold single atoms (0.05 and 0.3 wt.% Au) as very efficient catalysts that exhibited a high activity (>99.5% CO conversion), selectivity, and stability for PROX at PEMFC working temperature (Qiao et al., 2015). Developing such a type of catalyst was based on high CO oxidation activity and low reactivity toward hydrogen oxidation of single-atom catalysts in the temperature range of interest. It was demonstrated that modification of the active sites for CO oxidation could affect hydrogen oxidation, thus improving selectivity.

By combining two advantageous approaches, Reina et al. have prepared complex formulations to increase gold catalyst efficiency for PROX. These include (i) ceria monolayer dispersion on alumina to attain a higher surface-to-bulk ratio and (ii) promotion of redox properties and oxygen mobility of ceria by doping with various ions like La, Ni, Cu, Fe, Cr, and Y (Reina et al., 2015b). A beneficial effect of Fe and Cu on Au/CeO_2_/Al_2_O_3_ catalytic performance was observed and related to enhanced oxygen storage capacity. The role of iron as ceria promoter in gold-catalyzed PROX reaction has been studied in several papers (Laguna et al., 2010; Tabakova et al., 2011; Liao et al., 2012; Ilieva et al., 2013; Reina et al., 2016c). Variations of performance were ascribed to different preparation methods and doping amounts; however, in all cases, added iron affected ceria structural and electronic properties, in particular oxygen vacancies formation and gold dispersion. Additionally, depending on synthesis procedure, doping with iron caused a lower surface basicity, thus favoring stability in the presence of CO_2_. Cu-promoted samples combined the positive features of each active phase—high CO oxidation activity of Au/CeO_2_ at low temperatures and low activity of CuO_x_ for hydrogen oxidation—and were considered a promising alternative for H_2_ clean-up applications. Doping of ceria by small amounts of cobalt oxide was shown to improve the PROX oxidation activity in the presence of CO_2_ and H_2_O in the gas stream of series of catalysts based on Au/CeO_2_/Al_2_O_3_ (Reina et al., 2015c). Along with the promotional effect of cobalt oxide, some important questions have been raised that allow clarifying the PROX reaction, in particular support and gold nanoparticle function in hydrogen oxidation, and existence of a support-dependent hydrogen effect on CO oxidation. It was suggested that hydrogen oxidation could be used as a criterion for selection of suitable catalytic formulations for H_2_ clean-up reactions.

Various Cu content (0.24, 0.51, 1.04, and 1.55 wt.%) in Fe- and La-doped alumina supports has been used to study gold catalyst performance (Sun et al., 2016). A high dispersion of gold on copper-containing alumina-modified supports was reported. Simultaneous presence of Au and a higher copper loading improved catalyst activity, selectivity, and long-term stability; however, resistance to CO_2_ and water was not reported. In contrast to the advantages of Au–Cu bimetallic system, Liao et al. (2016) have reported decreasing CO conversion over Au/Al_2_O_3_ upon increasing copper amount by impregnation. TEM images and bimetallic particle size distribution diagrams of calcined samples showed an increased size after PROX. Deactivation at a higher reaction temperature was explained based on these findings. Reduction treatment in a flow of 50% H_2_/He at 300°C facilitated strong interaction between gold and copper due to copper mobility and Au–Cu nanoalloy formation. The average size of reduced catalyst particles remained the same after reaction; moreover, some stabilization of the gold nanoparticles on the alumina occurred.

**Figure 11:**
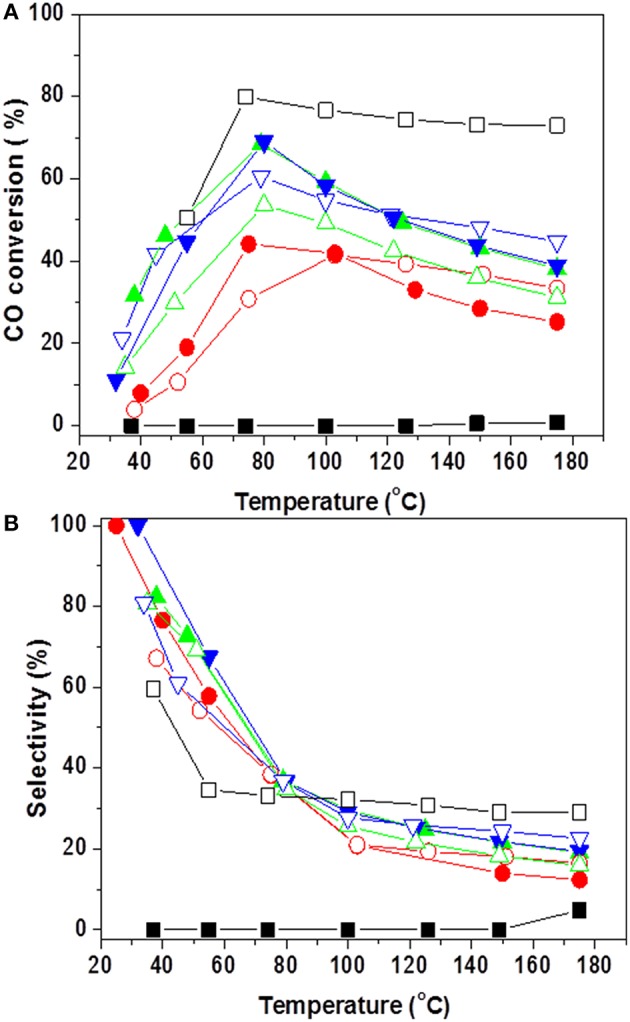
CO conversion **(A)** and selectivity **(B)** of gold catalysts on alumina-supported ceria (10, 20, or 30 wt.% CeO_2_), modified by Y_2_O_3_: AuAl - (■), AuCe - (□), AuCe10Al - (▿), AuYCe10Al-(▾), AuYCe20Al - (▵), AuYCe20Al - (▴), AuCe30Al-(O), AuYCe30Al-(●). Reprinted with permission from Ilieva et al. (2019). Copyright Elsevier B.V.

Looking for well-performing catalysts at a reasonable price, Ilieva et al. (2019) prepared alumina-supported ceria materials (10, 20, or 30 wt.% CeO_2_). A low content of Y_2_O_3_ (1 wt.% with respect to ceria amount) was applied and an improved performance was observed. Yttria amount was selected based on previous findings for promising performance of gold nanoparticles supported on Y-doped ceria in PROX reaction (Ilieva et al., 2016). In a detailed investigation of support preparation methods using yttria dopant, it has been shown that small amounts of Y_2_O_3_ exhibit a favorable effect on PROX activity and selectivity. By analyzing the important role of ceria surface modification and surface oxygen mobility, it was concluded that higher amounts of non-reducible dopant led to oxygen vacancies ordering and diminished CO conversion. Optimistic results in the presence of CO_2_ and water were reported for gold on alumina-supported Y-doped ceria (20 wt.% CeO_2_) by comparison with Au/CeO_2_ performance, the latter manifesting higher activity and selectivity at 100°C only by 10% (Figure 11). Also, the former catalyst, mostly composed of alumina (76 wt.%), exhibited good stability in the realistic post-reforming stream.

### Various Support Materials for Gold-Containing Catalysts

In this section, are summarized some examples of materials that are not typically employed in gold-catalyzed PROX reaction. Hexagonal mesoporous silica (HMS) was used as a support for gold catalysts, and the effect of modification by Fe^3+^, Ce^4+^, or Ti^4+^ ions on PROX performance was studied (Zapeda et al., 2010). All modified Au/HMS catalysts demonstrated higher activity and stability, with the Fe-promoted one performing very well. The best PROX activity and selectivity of this catalyst was attributed to its highest oxygen capacity, higher surface concentration of small gold particles (0.5–3.0 nm), and lowest catalyst deactivation caused by surface carbonate species. Laveille et al. (2016) have developed a durable catalyst for PROX by a new approach aiming to avoid catalyst deactivation by gold particle agglomeration and formation of surface carbonaceous species. Considering previous reports in the literature for some gold–support interface sites, in particular support surface oxygen and hydroxyl groups involved in these undesirable phenomena, the authors applied a one-pot reduction method to disperse gold nanoparticles (2.9 ± 1.2 nm) over the hydrophobic surface of commercial Aerosil R972 silica. Comparison of catalytic activity in PROX with that of gold on hydrophilic surfaces like Au/TiO_2_ and Au/Al_2_O_3_ pointed out a high long-term stability and selectivity of this new formulation.

**Figure 12:**
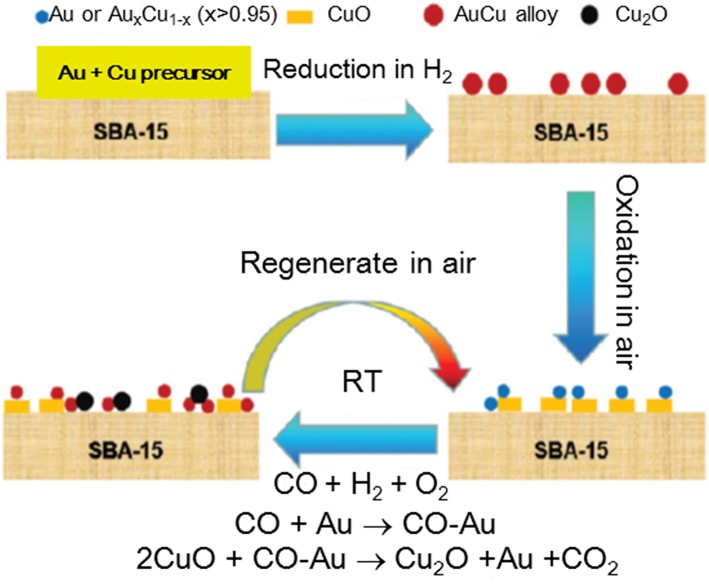
Graphic representation of catalyst reaction- deactivation mechanism. Reprinted with permission from Li et al. (2012), *ACS Catal*. 2 (3), 360–369. Copyright (2012) American Chemical Society.

By a nanoengineering technique, Li et al. (2012) have prepared a bimetallic catalyst on SBA-15, preliminary functionalized in order to facilitate high gold dispersion (average size of 3 nm) in close vicinity of well-dispersed CuO. Although high CO oxidation activity at room temperature was shown, this catalyst deactivated very fast at increased temperature due to Au–Cu nanoalloy formation in a hydrogen-rich gas mixture. Various techniques have been used to clarify structural changes after catalytic tests and activation procedure in air, allowing partial recovering of the PROX activity. A hypothesis for migration of metallic copper species into gold particles during the PROX and Cu reoxidation during the regeneration process provided some insights into the mechanism of activation and deactivation of this type of catalyst (Figure 12), but further investigations should address suitability and stability in realistic PROX feed.

Metallic gold colloids of well-defined particle size were deposited on inert amorphous silica or mesoporous SBA-15 modified by various titania amounts in order to assess the effect of Au–TiO_2_ perimeter length on the catalytic behavior (Beck et al., 2009). CO oxidation during PROX was significantly affected by the presence of hydrogen. A sample with the largest population of finely dispersed and low coordinated surface gold atoms exhibited the lowest selectivity due to domination of hydrogen oxidation at increasing temperature. Comparison of CO oxidation and PROX performance between Au/TiO_2_ and Au/TiO_2_/SiO_2_ catalysts showed that the CO oxidation was governed mainly by strong Au–TiO_2_ interface interaction, while the Au–SiO_2_ interface and gold particle size played a more important role during PROX.

### Gold Particle Size and PROX Performance

The size of supported gold particles is one of the most decisive factors in synthesis of active catalysts for various reactions. A general opinion exists that the abundance of small gold particles on the catalyst surface guarantees good performance. However, in the case of CO oxidation, one of the most studied gold-catalyzed reactions, Valden et al. (1998) have observed an optimum gold particle size over Au/TiO_2_ ranging from 2.5 to 3.0 nm due to quantum size effects with respect to gold particle thickness. Some of the above-commented works (Beck et al., 2009; Lakshmanan et al., 2016) focused on the relationship between performance for PROX and gold particle size. It was claimed that the presence of very small gold particles contributed not only to higher rates of CO conversion but also to unwanted hydrogen oxidation. Very recently, Qiu et al. (2019) explored the relationship between catalytic behavior in PROX and dispersion and oxidation states of active Au species in a series of Au/CeO_2_ catalysts with gold loading ranging within 0.05–1.2 wt.%. A higher CO oxidation activity was registered at a low temperature with an increase in gold amount up to 0.6 wt.%. A further increase in gold content gave rise to decreased CO conversion and selectivity. The presence of highly dispersed gold particles in samples of low gold content was suggested because it was difficult to observe any particles by HRTEM. However, an increased particle size was found at a higher gold loading, estimated to be about 4 nm in 0.6 wt.% Au/CeO_2_ and 10 nm in 1.2 wt.% Au/CeO_2_, thus explaining diminished activity. XPS analysis and *in situ* DRIFTS measurements allowed one to explain the best performance of low loaded Au/CeO_2_ (0.3 wt.% Au) with stabilization of Au^3+^ species by strong Au–support interaction. More insights into particle size effects on gold-catalyzed PROX reaction were gained by preparation of series of gold catalysts with well-defined gold nanocrystals of three different average sizes of about 2.1, 3.8, and 7.3 nm supported on CeO_2_, ZnO, and Al_2_O_3_ (Beck et al., 2018). Regardless of support type, samples with the smallest gold particles exhibited the highest reaction rates. The increase in size caused a drop of gold activity over all three supports. Based on the correlation between particle diameter and reaction rate, it was suggested that in the case of Au/CeO_2_, the most active species was perimeter corner sites consisting of low coordinated gold in close contact with ceria. According to previous literature report, corner and edge sites are the centers where hydrogen dissociation also proceeds (Bus et al., 2005). In view of this, the finding of Beck et al. for higher selectivity over samples of smaller gold particle size implies that depending on the experimental conditions, the CO oxidation could dominate over dissociative hydrogen adsorption. These very last findings confirm the importance of highly dispersed gold nanoparticles (2–3 nm) for the CO oxidation activity during PROX. However, the role of support, in particular suitable modification of Au–metal oxide perimeter sites (Hartadi et al., 2016) or optimum defect sites (Centeno et al., 2016), should also be considered in order to avoid selectivity decrease.

Concerning CO-PROX mechanism, Lakshmanan et al. (2014) have reviewed mechanistic aspects of the reaction by highlighting the role of the gold oxidation state and particle size, the nature of support, and the importance of the gold–support interface. Thereafter, in addition to already mentioned findings of Saavedra et al. (2016) and Whittaker et al. (2018), a significant contribution to mechanistic understanding of the PROX was made by the Behm group (Widmann et al., 2014; Wang et al., 2015; Hartadi et al., 2016). Special attention was paid to clarify the nature of active oxygen species for H_2_ oxidation and the interactions of CO and H_2_ with this oxygen species during the simultaneous presence of CO and H_2_ in the reaction mixture by applying a combination of characterization techniques including temporal analysis of product reactor measurements. In these papers, more details can be found because previous results and implications on PROX mechanism over supported Au catalysts are also summarized.

### Structured (Monolithic) Catalysts for PROX

The performance of gold-containing structured catalysts for PROX has been reported in a few papers. Moreno et al. (2016) have studied the activity and selectivity during PROX over ceramic monoliths wash-coated with a homogeneous layer of Au/TiO_2_ with well-dispersed gold nanoparticles at a wide range of operation conditions. The catalyst exhibited good CO oxidation activity (79% at 110°C), selectivity, and stability during 45 h on stream. A genetic algorithm approach was used for estimation of the reaction kinetic parameters of an empirical nonlinear model. Model results accurately reproduced the behavior of the PROX reaction. Metallic monoliths were already mentioned as an interesting alternative to ceramic monoliths. Anodized aluminum monoliths were wash-coated with Au/TiO_2_ by incipient wetness impregnation and hydrothermal synthesis and evaluated in PROX (Adrover et al., 2016). A CO conversion of about 60% and stability of CO activity and CO_2_ selectivity for at least 30 h operation were considered promising results, and an improved performance with higher catalyst loadings was suggested.

The use of microstructured reactors became an attractive research topic in the energy field of portable and mobile power generation. Recently, Kolb (2013) presented an overview of the achievements in the field of reactors for energy-related topics, reviewing also the PROX in microreactors. Divins et al. (2011) reported a new method for coating silicon microchannel walls with a thin, homogeneous, and well-adhered Au/TiO_2_ catalyst layer. Firstly, the silicon microchannels were functionalized by growing a SiO_2_ layer on the channel walls, then a TiO_2_ layer was formed, and, finally, pre-formed Au nanoparticles were anchored. Special attention was paid to the reactor design in order to eliminate heat from the highly exothermic CO oxidation reaction as a tool to affect selectivity and to avoid unwanted hydrogen oxidation. Silicon micromonolith performance was compared with that of conventional cordierite monolith wash-coated with the same catalyst. The tests demonstrated that silicon micromonoliths loaded with Au/TiO_2_ performed well in PROX, attaining about twofold higher specific activity values normalized per reactor volume amount when compared to conventional cordierite monoliths. Additionally, silicon micromonoliths outperformed cordierite monoliths in terms of stable operation without deactivation within 83 h in comparison with 48 h for conventional ones.

An Au/CuO_x_-CeO_2_ catalyst has been used in a microchannel reactor, and by applying computational fluid dynamics simulations, it was shown that the content of CO in realistic reformate stream, i.e., in the presence of CO_2_ and H_2_O, could be decreased below 100 ppm, and even to 10 ppm (Uriz et al., 2013). These levels of CO concentration were achieved in the temperature range of 155–175°C. An increase of microchannel characteristic size from 0.3 to 2.8 mm negatively affected the CO oxidation efficiency because of the stronger detrimental effect of the mass transport limitations on the CO oxidation than that on the H_2_ oxidation. Similarly as commented above in the case of silicon micromonolith, the desired removal of CO could be achieved by careful control of PROX reactor cooling by air and inlet temperature.

## Other Approaches to Gold-Based Catalysts and H_2_ Clean-Up Process Intensification

Advantages of the “supported approach” for preparation of efficient WGS catalysts were noted in Section The Application of “Supported Approach” based on a study of structure–WGS reactivity relationship of gold supported on CuO–CeO_2_ or alumina-supported CuO–CeO_2_ mixed oxides (Reina et al., 2016b). Previous works about CuO–CeO_2_ applicability in WGS and PROX reactions as well as the role of small amounts of gold to promote both WGS and PROX extended the knowledge of catalyst performance in these reactions as two steps in one attractive hydrogen purification process. The beneficial role of gold introduction was well-demonstrated in the case of alumina-supported materials, thus implying effectiveness of the multicomponent systems for CO clean-up *via* WGS and PROX. Based on the total amount of active phase in a catalyst formulation, calculations of specific activities provide further insights into superiority of these new catalysts from both economic and environmental points of view.

In view of H_2_ clean-up process intensification, a relevant study has been performed by Reina et al. to assess possibilities of conducting WGS and PROX reactions in a single reactor using gold-based catalysts (Reina et al., 2014b). Such a reactor design is expected to reduce costs and system volume that are desired features in the case of portable applications. However, the starting point to realize this idea is to develop a highly effective catalyst for both reactions. Gold-based catalysts were prepared using the direct anionic exchange method on alumina-supported Ce–Fe mixed oxides with properly selected iron oxide content to guarantee formation of a CeO_2_-FeO_x_ solid solution. In this way, the replacement of Ce^4+^ by Fe^3+^ favors oxygen mobility and facilitates high CO oxidation activity. Detailed analysis of the effect of operating conditions of both processes (temperature interval, space velocity, and composition of gas mixtures) indicated that by careful tuning of the reaction parameters, it is possible to achieve successful implementation of both reactions using one multipurpose catalyst.

## Concluding Remarks

This review summarizes recent advances in design of gold-based catalysts for H_2_ clean-up reactions focusing on different approaches to developing highly efficient and low-cost catalytic materials. On dispersing catalytic active components over high surface area supports such as alumina, silica, etc., a successful strategy was demonstrated for achievement of higher surface-to-bulk ratios, thus contributing to preparation of high-performing gold catalysts. A new trend is the usage of more complex compositions and structures prepared by different synthesis procedures. Detailed examination of atomically dispersed supported gold catalysts reveals their potential application due to the opportunity of maximizing gold atom efficiency and minimizing costs. The processes and catalysts for small-scale applications such as residential fuel cells or on-board hydrogen generators pose some challenging requirements that gold-based structured catalysts and microchannel reactors could comply. During the last years, various types of durability tests of gold/metal oxide catalysts under realistic reaction conditions became an important part of the investigations. Many researchers consider both high gold dispersion and appropriate support modifications as strategies to achieve higher activity and stability as well as higher selectivity in the case of PROX. New insights into WGS and PROX reaction mechanisms and the nature of active sites have been gained from theoretical studies and exploration of model catalysts. In the last decade, a remarkable progress has been made in elucidating various aspects of gold-catalyzed reactions for clean hydrogen production, in particular WGS and PROX reactions, by using sophisticated *in situ* and *in operando* characterization techniques. Many comprehensive studies greatly boost the development of highly efficient gold-based catalysts and contribute to successful dealing with environmental and energy concerns that world is facing. The review could serve as a good basis for future investigations, because gold catalysis is a field with huge potential. Despite all noted achievements, the structure/composition–catalytic activity relationship and methods for improving performance will continue to attract the scientific interest hoping to understand as much as possible the mysterious nature of the gold catalysts.

## Author Contributions

The author confirms being the sole contributor of this work and has approved it for publication.

### Conflict of Interest Statement

The author declares that the research was conducted in the absence of any commercial or financial relationships that could be construed as a potential conflict of interest.
